# MicroRNA-145-5p modulates Krüppel-like factor 5 and inhibits cell proliferation, migration, and invasion in nasopharyngeal carcinoma

**DOI:** 10.1186/s12860-022-00430-9

**Published:** 2022-07-14

**Authors:** Chien-Han Yuan, Wei-Chi Hsu, A.-Mei Huang, Ben-Chih Yuan, I.-Hung Chen, Chia-An Hsu, Rong-Feng Chen, Yih-Min Chu, Hui-Hui Lin, Hung-Lung Ke

**Affiliations:** 1 Department of Otolaryngology, Kaohsiung Armed Forces General Hospital, Kaohsiung, Taiwan; 2 Institute of Medical Science and Technology, National Sun Yat-Sen University, Kaohsiung, Taiwan; 3 Department of Otolaryngology, National Defense Medical Center, Taipei, Taiwan; 4 Department of Urology, School of Medicine, College of Medicine, Kaohsiung Medical University, Kaohsiung, Taiwan; 5 Graduate Institute of Medicine, College of Medicine, Kaohsiung Medical University, Kaohsiung, Taiwan; 6 Graduate Institute of Clinical Medicine, College of Medicine, Kaohsiung Medical University, Kaohsiung, Taiwan; 7 Department of Biochemistry, School of Medicine, College of Medicine, Kaohsiung Medical University, Kaohsiung, Taiwan; 8 Department of Otolaryngology, Fooyin University Hospital, Pingtung, Taiwan; 9 Department of Internal Medicine, Pingtung Branch, Kaohsiung Armed Forces General Hospital, Pingtung, Taiwan; 10 Department of Urology, Kaohsiung Medical University Hospital, Kaohsiung Medical University, Kaohsiung, Taiwan; 11 Department of Urology, Kaohsiung Municipal Ta-Tung Hospital, Kaohsiung Medical University Hospital, Kaohsiung Medical University, Kaohsiung, Taiwan; 12 Department of Business Management, National Sun Yat-sen University, Kaohsiung, Taiwan

**Keywords:** Nasopharyngeal carcinoma, MicroRNA-145-5p, Krüppel-like factor 5, Focal adhesion kinase, Cell migration, Cell invasion

## Abstract

**Background:**

In several human cancers, Krüppel-like factor 5 (KLF5), a zinc finger transcription factor, can contribute to both tumor progression or suppression; however, the precise role of KLF5 in nasopharyngeal carcinoma (NPC) remains poorly understood. In this study, the association between KLF5 and microRNA-145-5p (miR-145-5p) in NPC cells was elucidated.

**Results:**

Our results showed that KLF5 expression was up-regulated in NPC group compared to normal group. We found that KLF5 exhibited an oncogenic role in NPC cells. The upregulation of miR-145-5p inhibited the proliferation, migration, and invasion of NPC cells. It was observed that miR-145-5p could down-regulate the mRNA and protein expression of KLF5 in NPC cell lines. Additionally, the activity of focal adhesion kinase (FAK), a migration marker, was regulated by miR-145-5p and KLF5 in NPC cells.

**Conclusions:**

The results of this study indicated that miR-145-5p could repress the proliferation, migration, and invasion of NPC cells via KLF5/FAK regulation, and could be a potential therapeutic target for patients with NPC.

**Supplementary Information:**

The online version contains supplementary material available at 10.1186/s12860-022-00430-9.

## Background

Nasopharyngeal carcinoma (NPC), which develops in the nasopharyngeal epithelium, is one of the most common squamous cell carcinomas of the head and neck in North Africa and Southeast Asia but are rare in Western countries [1]. Compared to the other head and neck cancers, metastasis of the neck lymph node and distant metastasis are commonly observed in NPC [2], making its treatment challenging. The long-term survival rate of patients with NPC has improved with the advancement in intensity-modulated radiation therapy and adjuvant chemotherapy; however, local relapse and distant metastasis continue to remain the leading causes of mortality [3, 4]. In Taiwan, according to the Cancer Registry Annual Report, which was published by the Health Promotion Administration Ministry of Health and Welfare in December 2018, there were 1,518 new cases of NPC and 678 deaths due to NPC in 2016. Therefore, the molecular mechanisms underlying the tumorigenesis and malignant progression of NPC needs to be elucidated to aid in the development of effective diagnostic and therapeutic tools.

Krüppel-like factor 5 (KLF5) is a transcription factor containing three highly conserved zinc finger domains at its C-terminus, which bind to CACC or GC boxes and regulate downstream genes, such as p27, NOTCH1, VEGFA, SOX4, and Cyclin D1 [5, 6]. KLF5 has been found to participate in several biological functions, such as cell proliferation, migration, autophagy, and apoptosis. KLF5 also plays an important role in disease development, especially in cancers. KLF5 has an oncogenic role in certain types of cancer, including pancreatic, colorectal, breast,  and cervical cancer [7–10]. However, the detailed underlying molecular mechanisms and role of KLF5 in NPC has not yet been explored [11].

MicroRNAs (miRNAs) are 22–24 nucleotide-long non-coding RNAs that regulate gene expression by targeting the 3'-UTR of the target mRNA [12]. Several human tumors and other malignancies are correlated with altered and abnormal miRNA expression, respectively [13]. Several miRNAs participate in NPC pathogenesis through the alteration of gene networks [14]. Some miRNAs have been found to regulate KLF5 in certain cancers. In osteosarcoma, miR‐493‐5p represses cell metastasis and proliferation by targeting KLF5 [15]. The inhibition of KLF5 by miR‑153 suppresses cellular invasion in laryngeal squamous cell carcinoma [16], while miR-4711-5p reduces KLF5 mRNA and protein expression in colon cancer cells [17]. However, the molecular mechanisms underlying the association between miRNAs and KLF5 in NPC are unknown. In NPC, miR-145-5p, a well-known tumor suppressor miRNA, is downregulated [14, 18]. Several studies have found that miR-145-5p can regulate KLF5 expression in various cancers, such as cervical cancer, hepatocellular carcinoma, and gastric cancer [19–21].

In this study, we aimed to elucidate the association between miR-145-5p and KLF5 and its effect on NPC development.

## Results

### KLF5 overexpression modulates the proliferation, migration, and invasion of NPC cells

**Fig. 1:**
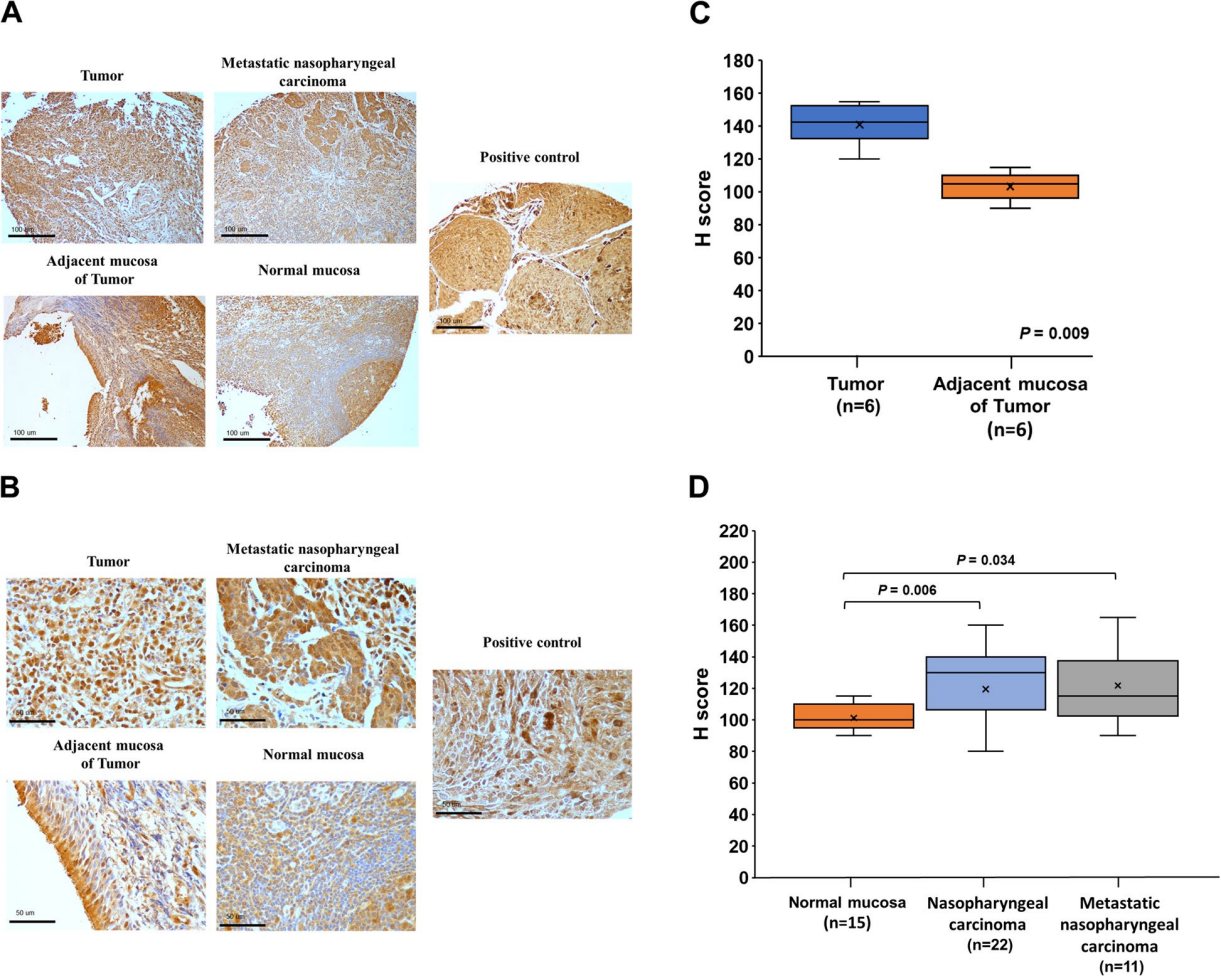
KLF5 expression is up-regulated in nasopharyngeal carcinoma (NPC) tissues. **a, b** Representative immunohistochemistry images of KLF5 in nasopharyngeal carcinoma tissues, adjacent mucosa of tumor tissues, metastatic nasopharyngeal carcinoma tissues and normal mucosa tissues. **c** Boxplot depicting KLF5 H-score distribution in paired tissues with adjacent mucosa (*n* = 6) and nasopharyngeal carcinoma (*n* = 6). **d** Boxplot depicting KLF5 H-score distribution in normal mucosa (*n* = 15), nasopharyngeal carcinoma (*n* = 22) and metastatic nasopharyngeal carcinoma (*n* = 11). Magnification, × 200; scale bar, 100 µm. Magnification, × 400; scale bar, 50 µm

**Fig. 2:**
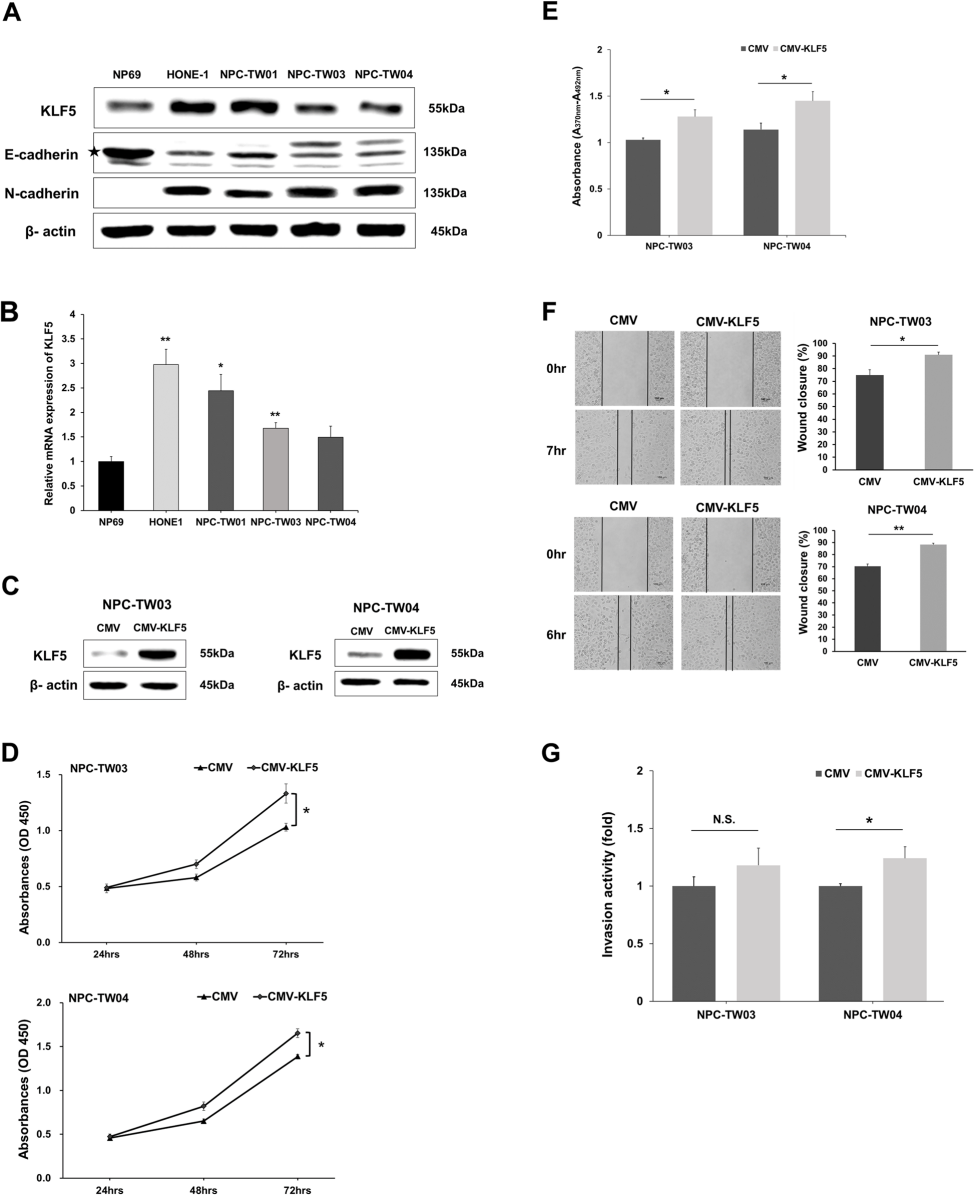
KLF5 overexpression mediates the proliferation, migration, and invasion of nasopharyngeal carcinoma (NPC) cells. **a** Western blot analysis indicating the protein expression of KLF5, E-cadherin and N-cadherin in NP69, HONE-1, NPC-TW01, NPC-TW03, and NPC-TW04 cells, were probes with anti KLF5, E-cadherin and N-cadherin and specific antibodies for each compartments corresponding to anti β-actin. **b** Real-time PCR results showing the mRNA expression of KLF5 in NP69 and NPC cell lines using GAPDH as a reference gene. **c** Western blot analysis indicating the expression of KLF5 in NPC-TW03 and NPC-TW04 cells after CMV or CMV-KLF5 vector transfection, were probes with anti KLF5 and specific antibodies for each compartments corresponding to anti β-actin. **d** Proliferation rates of NPC cells 24 to 72 h post-transfection with CMV or CMV-KLF5. **e** Proliferation rates of NPC cells at 72 h post-transfection with CMV or CMV-KLF5 was analyzed by BrdU ELISA cell proliferation assay. **f** NPC-TW03 and NPC-TW04 cells were transiently transfected with CMV or CMV-KLF5 and analyzed by wound healing assays. **g** NPC-TW03 and NPC-TW04 cells were transiently transfected with CMV and CMV-KLF5 and analyzed by invasion assays. Data are shown as mean ± SD from three independent experiments. *P < 0.05, **P < 0.01, respectively. N.S., not significant

In immunohistochemistry staining of KLF5 (Fig. 1a and b), we found KLF5 protein expression was higher in nasopharyngeal carcinoma tissues in comparison to that of paired adjacent mucosa tissues (Fig. 1c). In addition, nasopharyngeal carcinoma and metastatic nasopharyngeal carcinoma tissues also had higher KLF5 expression levels than normal mucosa (Fig. 1d). Next, we used four NPC cell lines and one nasopharyngeal epithelial cell line for an in vitro analysis. As no studies compare the cell characteristic between NP69 and four NPC cells, we used western blot analysis and found that the protein expression of E-cadherin was higher and N-cadherin was lower in NP69 nasopharyngeal epithelial cell line than four NPC cell lines (Fig. 2a). These data indicated that NP69 were epithelial-like cells, and four NPC cells inclined to mesenchymal-like cells. In addition, we found that HONE-1 and NPC-TW01 cells had higher protein levels of KLF5 than NP69, NPC-TW03 and NPC-TW04 cells (Fig. 2a). Real-time PCR analysis revealed that NP69 cells had lower KLF5 mRNA expression than four NPC cells (Fig. 2b). In order to identify the role of KLF5 in the development of NPC, KLF5 was overexpressed by transfecting vectors, CMV-KLF5, in NPC-TW03 and NPC-TW04 cell lines (Fig. 2c). The overexpression of KLF5 resulted in proliferation promotion of NPC-TW03 and NPC-TW04 cells (Fig. 2d and e). Wound healing assay confirmed that the overexpression of KLF5 resulted in the promotion of wound closure in NPC-TW03 and NPC-TW04 cell migration (Fig. 2f). The cell invasion assay results showed that KLF5 overexpression increased invasive ability in NPC-TW04 cells (Fig. 2g). These results indicated that KLF5 may play an oncogenic role in NPC cells by regulating the proliferation, migration, and invasion of NPC cells.

### MiR-145-5p suppresses the proliferation, migration, and invasion of NPC cell lines

**Fig. 3:**
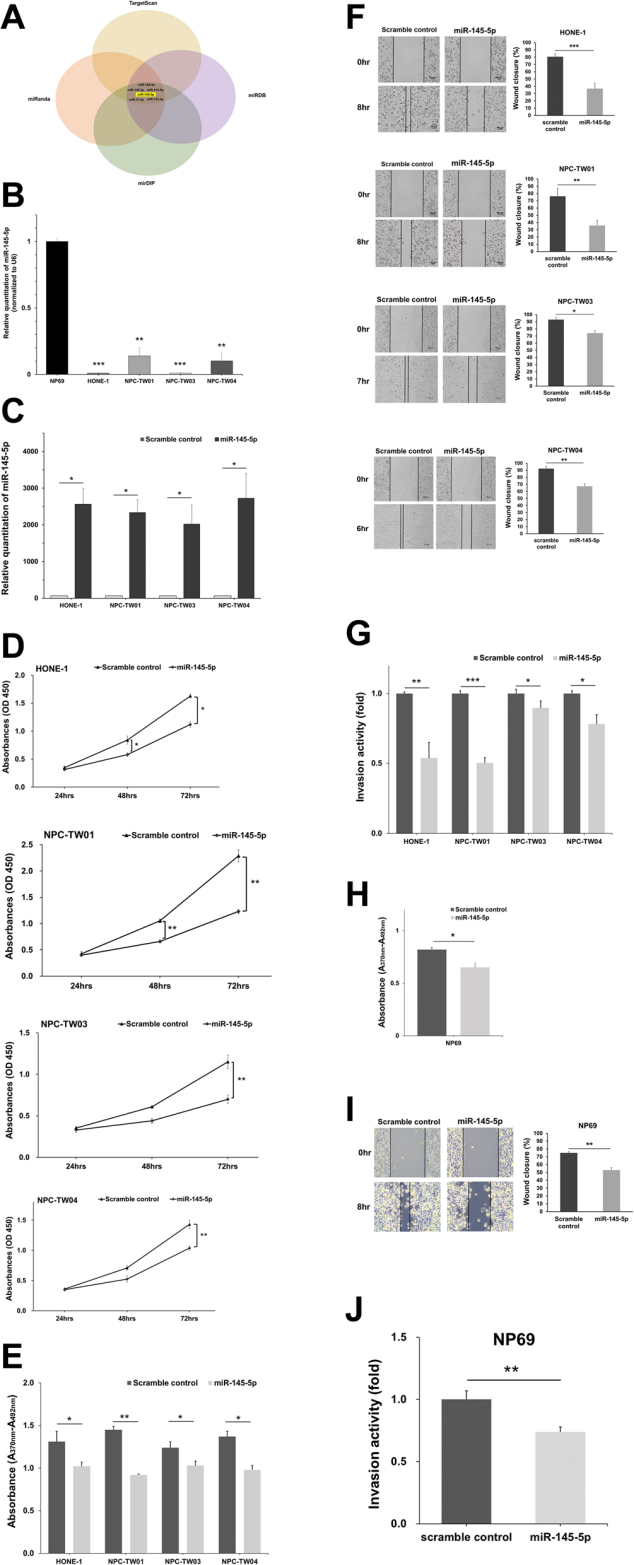
MiR-145-5p represses the proliferation, migration, and invasion of nasopharyngeal carcinoma (NPC) cell lines. **a** A schematic illustration describing that putative miR-143-3p, miR-148-3p, miR-145-5p, miR-153-3p, miR-21-5p and miR-214-5p  might directly target KLF5. Data was obtained and analyzed from four prediction databases: TargetScan, miRDB, mirDIP, and miRanda. **b** Real-time PCR results showing the expression of miR-145-5p in NP69 and four NPC cell lines. **c** Real-time PCR results indicating miR-145-5p expression in four NPC cells transfected with scramble control or miR-145-5p mimics. **d** Proliferation rates of NPC cells 24 to 72 h post-transfection with a scramble control or miR-145-5p mimics. **e** Proliferation rates of NPC cells at 72 h post-transfection with a scramble control or miR-145-5p mimics was analyzed by BrdU ELISA cell proliferation assay. **f** NPC cells were transiently transfected with scramble control and miR-145-5p mimics and analyzed by wound healing assays. **g** NPC cells were transiently transfected with scramble control and miR-145-5p mimics and analyzed by invasion assays. **h** Proliferation rates of NP69 cells at 72 h post-transfection with a scramble control or miR-145-5p mimics was analyzed by BrdU ELISA cell proliferation assay. **i** NP69 cells were transiently transfected with scramble control and miR-145-5p mimics and analyzed by wound healing assays. **j** NP69 cells were transiently transfected with scramble control and miR-145-5p mimics and analyzed by invasion assays. Data are shown as mean ± SD from three independent experiments. *P < 0.05, **P < 0.01, ***P < 0.001, respectively

In order to elucidate the molecular mechanism underlying the modulatory activity of KLF5 on the function of NPC cells, we examined the possible upstream miRNA regulators of KLF5 using four different miRNA target-predicting tools, and we found miR-143-3p, miR-148-3p, miR-145-5p, miR-153-3p, miR-21-5p and miR-214-5p may target to 3'-UTR of KLF5 mRNA. MiR-145-5p is a well-known tumor suppressor microRNA in many cancers; however, the role and molecular mechanism of miR-145-5p in nasopharyngeal carcinoma is still unknown, so we finally selected miR-145-5p as the possible candidate miRNA (Fig. 3a). We analyzed miR-145-5p expression in vitro. The data showed that miR-145-5p expression levels were lower in all four NPC cells, compared to NP69 cells (Fig. 3b). In order to evaluate the biological function of miR-145-5p in NPC cells, we examined cellular proliferation, migration, and invasion by transfecting with miR-145-5p mimics in these four NPC cell lines (Fig. 3c). As depicted in Fig. 3d and e, the overexpression of miR-145-5p inhibited the proliferation of NPC cells, compared to that of the scrambled control. MiR-145-5p effectively repressed the migration and invasion of four NPC cell lines, in comparison to that of the control cells (Fig. 3f and g). Furthermore, increased miR-145-5p expression also repressed the proliferation, migration, and invasion ability of NP69 cells (Fig. 3h ~ j).

### KLF5 expression is modulated by miR-145-5p in NPC cells

**Fig. 4:**
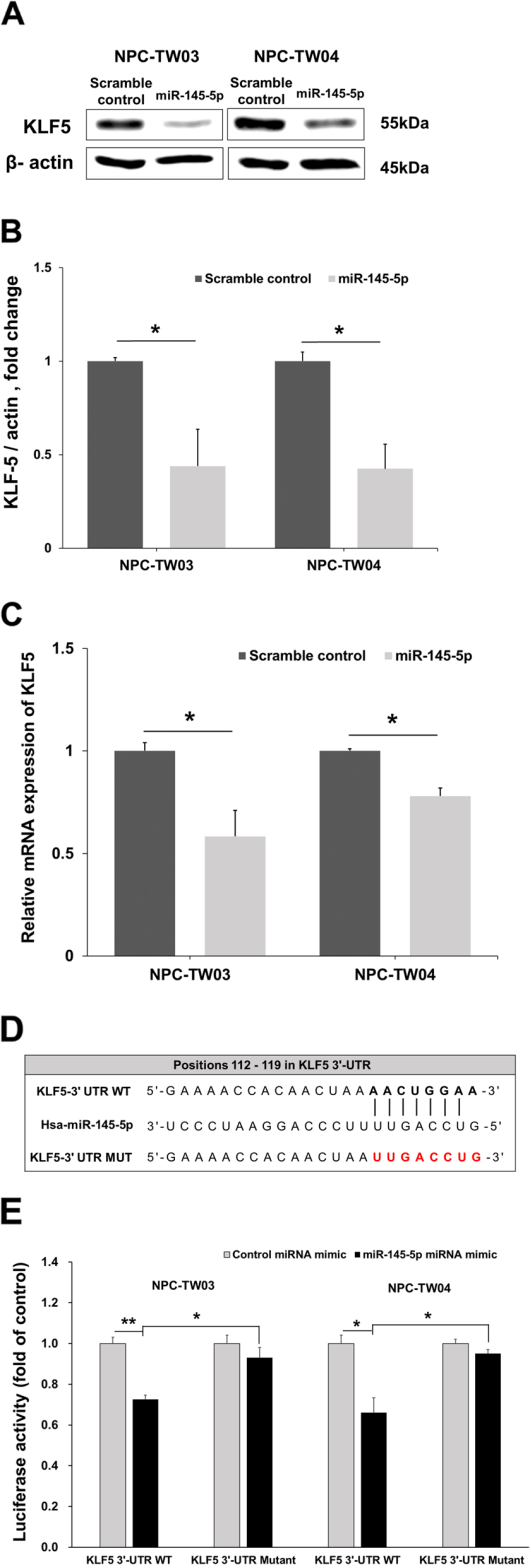
MiR-145-5p regulates KLF5 protein expression. **a** Western blot analysis of KLF5 levels in NPC-TW03 and NPC-TW04 cells post-transfection with scramble control or miR-145-5p mimics, were probes with anti KLF5 and specific antibodies for each compartments corresponding to anti β-actin. **b** Quantification of KLF5 expression are the average of three independent experiments, using the Image J software using β-actin as a reference. **c** MiR-145-5p down-regulates KLF5 mRNA expression after scramble control or miR-145-5p mimics transfection in NPC-TW03 and NPC-TW04 cells. **d** Complementarity between the seed sequence of hsa-miR-145-5p and the targeted sequence in the 3'-UTR of KLF5 mRNA predicted by TargetScan. **e** Relative luciferase activity of wild-type (WT) and mutated KLF5 3'-UTR luciferase reporters in NPC-TW03 and NPC-TW04 cells after miR-145-5p mimics or scramble control co-transfection. Data are shown as mean ± SD from three independent experiments. *P < 0.05, **P < 0.01, respectively

The association between KLF5 and miR-145-5p was further validated by examining the endogenous levels of the KLF5 in NPC cells following transfection with miR-145-5p mimics. Western blot analysis revealed that the miR-145-5p mimics reduced KLF5 expression in NPC-TW03 and NPC-TW04 cells (Fig. 4a and b). Real-time PCR results revealed that the mRNA expression of KLF5 was downregulated in NPC-TW03 and NPC-TW04 cells after transfection with miR-145-5p mimics (Fig. 4c). The 3'-UTR of the KLF5 mRNA contains a predicted binding site for miR-145-5p (Fig. 4d). Furthermore, we used dual-luciferase reporter assays to analyze whether miR-145-5p directly targeted KLF5. The reporter with the wild-type KLF5 3'-UTR showed decreased luciferase activity in NPC-TW03 and NPC-TW04 cells following transfection with miR-145-5p mimics, and was reversed in the presence of mutated KLF5 3'-UTR in comparison to that of the scrambled control (Fig. 4e). These findings suggest that miR-145-5p regulated the expression of KLF5 by direct target to its 3'-UTR.

### MiR-145-5p and KLF5 regulate the activity of focal adhesion kinase (FAK) in NPC cell lines

**Fig. 5:**
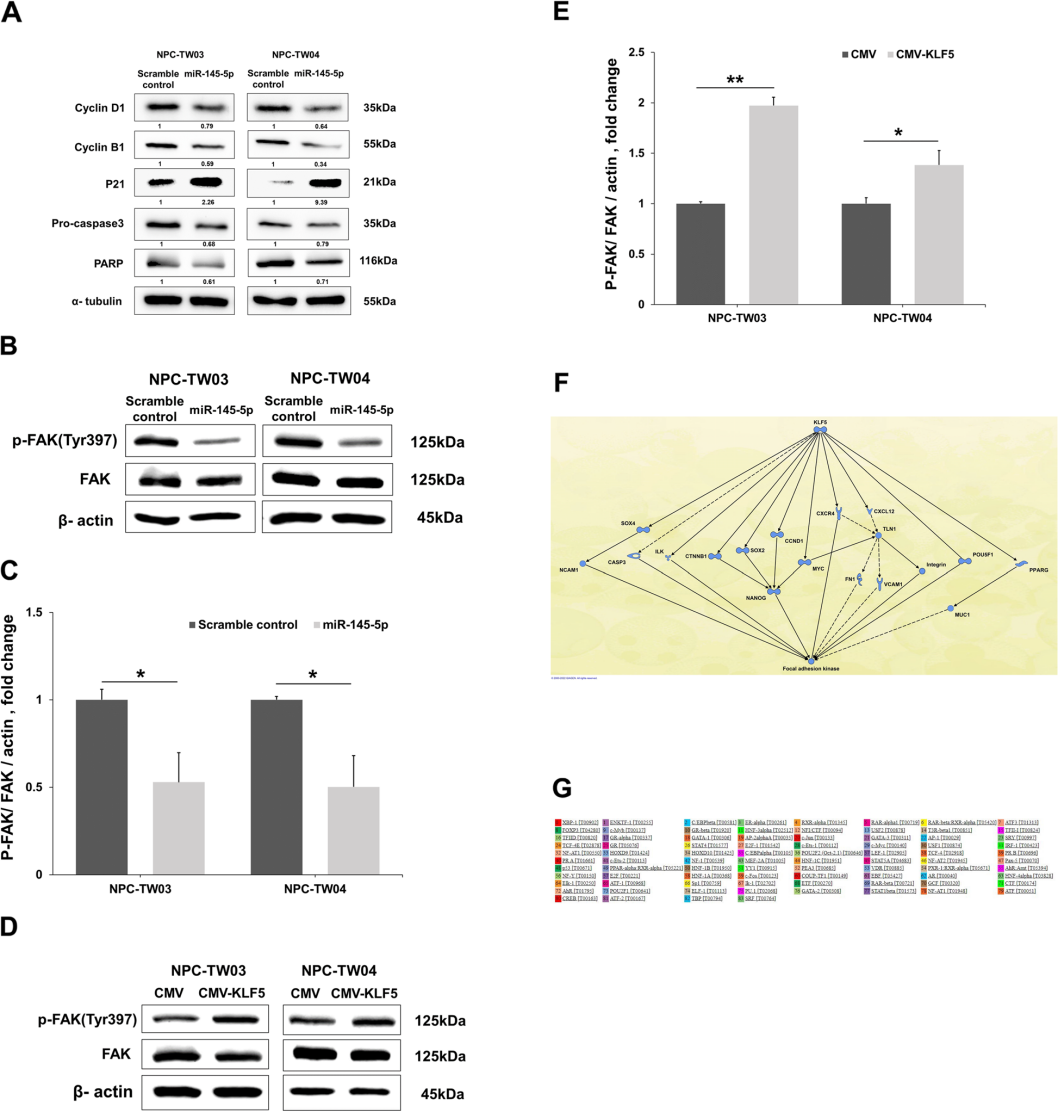
MiR-145-5p and KLF5 modulates phospho-FAK^Tyr397^ expression in nasopharyngeal carcinoma (NPC) cells. **a** MiR-145-5p induced p21 and reduced cyclin D1, cyclin B1, pro-caspase 3 and PARP protein expression post-transfection with scramble control or miR-145-5p mimics in NPC-TW03 and NPC-TW04 cells, were probes with anti cyclin D1, cyclin B1, p21, pro-caspase 3, PARP and specific antibodies for each compartments corresponding to anti α-tubulin. **b** MiR-145-5p down-regulates phospho-FAK^Tyr397^ protein expression post-transfection with scramble control or miR-145-5p mimics in NPC-TW03 and NPC-TW04 cells, were probes with anti phospho-FAK^Tyr397^, FAK and specific antibodies for each compartments corresponding to anti β-actin. **c** Quantification of phospho-FAK^Tyr397^/FAK expression are the average of three independent experiments, using the Image J software and β-actin as a reference. **d** Western blot analysis indicating phospho-FAK^Tyr397^ and FAK expression in NPC-TW03 and NPC-TW04 cells post-transfection with CMV or CMV-KLF5 vectors, were probes with anti phospho-FAK^Tyr397^, FAK and specific antibodies for each compartments corresponding to anti β-actin. **e** Quantification of phospho-FAK^Tyr397^/FAK expression are the average of three independent experiments, using the Image J software and β-actin as a reference. **f** The possible molecular network between KLF5 and FAK was analyzed by Ingenuity Pathway Analysis. **g** PROMO tool identified the putative transcription factor binding sites in FAK DNA sequences. Data are shown as mean ± SD from three independent experiments. *P < 0.05, **P < 0.01, respectively

Western blot analysis showed that the overexpression of miR-145-5p increased p21 and decreased cyclin D1, cyclin B1, pro-caspase3 and full length PARP protein levels in NPC-TW03 and NPC-TW04 cells (Fig. 5a). These data suggest that miR-145-5p could alter cell cycle progression and survival markers. Previous studies have demonstrated that FAK signaling plays a crucial role in modulating cell proliferation, migration, and invasion in NPC [22, 23]. In order to elucidate the mechanism by which miR-145-5p and KLF5 affects cellular function in NPC, the molecules related to the activation of FAK were analyzed. Western blot analysis revealed that the overexpression of miR-145-5p suppressed the activation of FAK in NPC-TW03 and NPC-TW04 cells (Fig. 5b and c). In addition, we found that KLF5 overexpression induced FAK activity in NPC-TW03 and NPC-TW04 cells (Fig. 5d and e). Using Ingenuity Pathway Analysis (IPA), we identified potential molecular networks between KLF5 and FAK (Fig. 5f). Furthermore, we used PROMO tool to identify the putative transcription factor binding sites in FAK DNA sequences; however, FAK didn’t have KLF5 transcription factor binding sites (Fig. 5g). We considered that KLF5 may indirectly altered FAK activity in NPC cells. These results suggest that the effect of miR-145-5p on cellular proliferation, migration and invasion in NPC might be mediated by the KLF5/FAK pathway.

### ML264 inhibits cell proliferation, migration, invasion and FAK activity in NPC cell lines

**Fig. 6:**
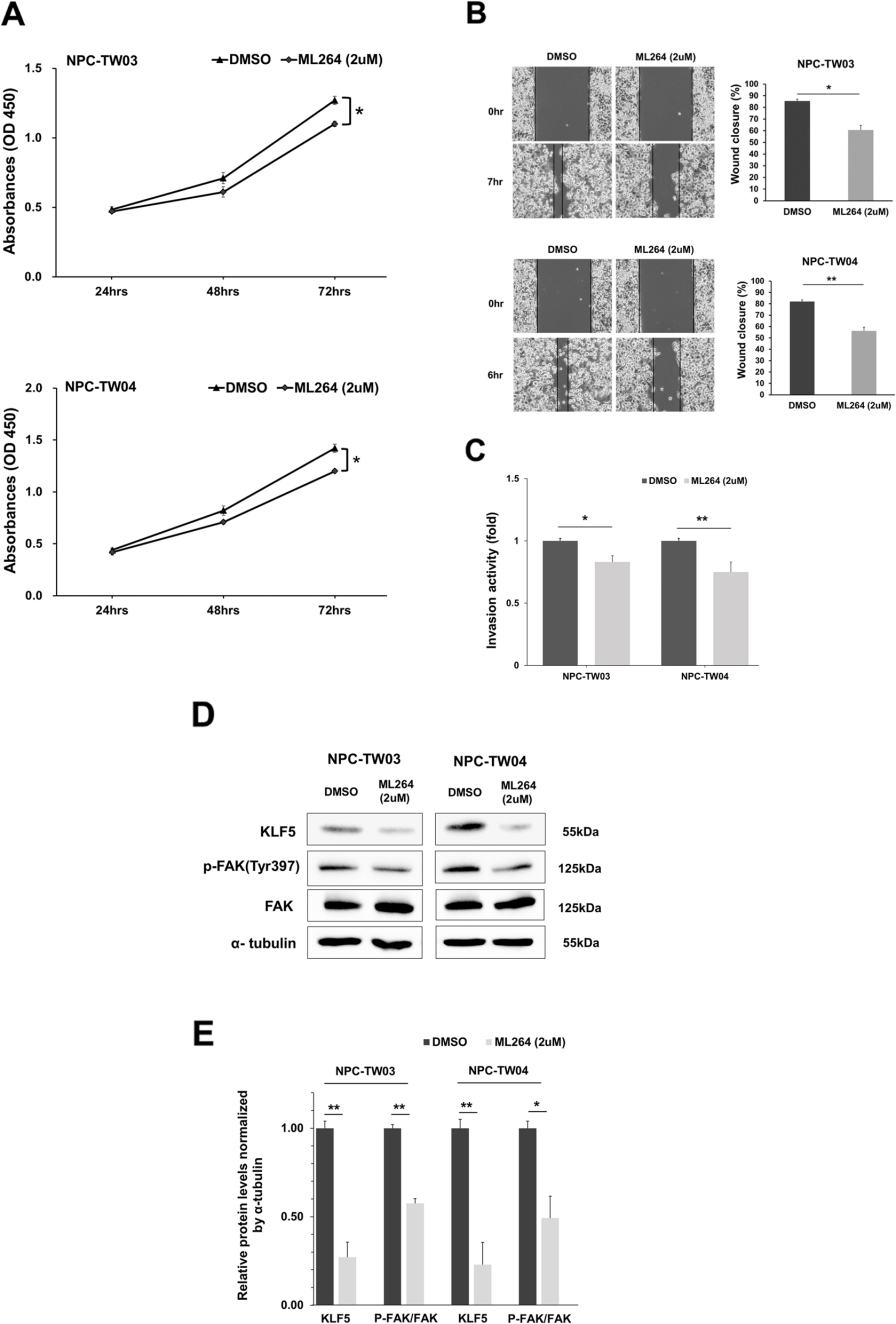
ML264 suppresses NPC-TW03 and NPC-TW04 cell proliferation, migration, invasion and FAK activity. **a** Proliferation rates of NPC-TW03 and NPC-TW04 cells 24 to 72 h post-treatment with DMSO or ML264 (2uM). **b** NPC-TW03 and NPC-TW04 cells were treated with DMSO or ML264 (2uM) and analyzed by wound healing assays. **c** NPC-TW03 and NPC-TW04 cells were treated with DMSO or ML264 (2uM) and analyzed by invasion assays. **d** Western blot analysis of KLF5, phospho-FAK^Tyr397^ and FAK levels in NPC-TW03 and NPC-TW04 cells treatment with DMSO or ML264 (2uM) for 24 h, were probes with anti KLF5, phospho-FAK^Tyr397^, FAK and specific antibodies for each compartments corresponding to anti α-tubulin. **e** Quantification of KLF5 and phospho-FAK^Tyr397^ /FAK expression are the average of three independent experiments, using the Image J software and α-tubulin as a reference. Data are shown as mean ± SD from three independent experiments. *P < 0.05, **P < 0.01, respectively

ML264 is a small molecule compound that specific represses the expression of KLF5. Although ML264 inhibits cell growth in colorectal and osteosarcoma cancer cells [24, 25], the effects of its suppression have not been explored in nasopharyngeal carcinoma. NPC-TW03 and NPC-TW04 cells were treated with ML264 (2uM) for 24, 48, and 72 h, after which cell viability was tested using the WST-1 reagent. ML264 treatment suppressed cell proliferation in NPC-TW03 and NPC-TW04 cells (Fig. 6a). In addition, ML264 also inhibited cell migration and invasion of NPC-TW03 and NPC-TW04 (Fig. 6b and c). Importantly, Western blot analysis showed that ML264 significantly reduced KLF5 protein expression and FAK activity in NPC-TW03 and NPC-TW04 cells (Fig. 6d and e). These results suggest that ML264 treatment was able to repress the proliferative, migrative, invasive capacity and FAK activity of NPC cells.

## Discussion

The results of the present study demonstrated that the upregulation of KLF5 expression modulated the function of NPC cells. The overexpression of miR-145-5p down-regulated the mRNA and protein expression of KLF5, and inhibited the proliferation, migration, and invasion of the NPC cell lines. Our study also revealed that miR-145-5p suppresses the activity of FAK.

In humans, the KLF5 protein, also known as IKLF or BTEB2, is an important member of the KLF family. KLF5 has opposing roles and either acts as a transcriptional repressor or activator of cellular function, or as a regulator in numerous tumors [26]. KLF5 has been verified to act as an oncogene in gastric cancer, breast cancer, leukemia, bladder cancer, and ovarian cancer. It has been demonstrated that KLF5 acts as a tumor suppressor in prostate cancer, esophageal cancer, and pituitary adenoma [27–29]. In this study, we found that overexpression of KLF5 can exert an oncogenic role in NPC cells to regulate cell proliferation, migration, and invasion. Furthermore, we found ML264, was a third-generation KLF5 inhibitor could inhibit NPC cell proliferation, migration, invasion and FAK activity. Therefore, ML264 might be a potential therapeutic agent to target nasopharyngeal carcinoma.

Certain microRNAs have been reported to modulate the development and progression of NPC [30, 31]. In this study, we examined the role of miR-145-5p, which is downregulated in several cancers, including NPC [13, 32]. We verified that miR-145-5p plays a significant role in the proliferation, migration, and invasion of NPC cells. These results are consistent with those from previous studies reporting the role of miR‐145‐5p as a tumor suppressor microRNA in NPC [33, 34]. Furthermore, we found that overexpression of miR-145-5p in NP69 cells also inhibited cell proliferation, migration and invasion. NP69 cells were an immortalized nasopharyngeal epithelial cell line while possessing many of the genetic signatures observed in NPC [35]. This cell line was established from primary nonmalignant nasopharyngeal epithelial cells and may represent a model of premalignant nasopharyngeal epithelial cells. We considered that miR-145-5p upregulation may prevent nonmalignant nasopharyngeal epithelial cells transformed into cancerous cells, and it needs to be verified by more exploration in the future.

KLF5 mRNA could be regulated by non-coding RNAs, such as microRNAs and long non-coding RNAs (lncRNAs) [36]. By using microRNA target-predicting tools, we identified miR-145-5p as a possible microRNA candidate that directly targets KLF5. The dual-luciferase assay results showed that miR-145-5p directly targets the 3'-UTR of KLF5 mRNA in NPC-TW03 and NPC-TW04 cells. We also used miRBase to observe whether any microRNAs would bind to the promoter of KLF5. We found 18 microRNAs may bind to KLF5 promoter, but not including miR-145-5p. Furthermore, KLF5 proteins regulated by certain post-translational modifications, including ubiquitination, phosphorylation, SUMOylation and acetylation [6]. In previous studies, miR-145-5p suppresses KLF5 expression in gastric cancer and hepatocellular carcinoma [20, 21]. Our results demonstrated that the overexpression of miR-145-5p could down-regulate the levels of KLF5 protein in NPC cells. The KLF5 protein is regulated through proteasome degradation after ubiquitination by E3 ubiquitin ligases, such as WWP1, F-box and WD repeat domain-containing 7 (FBW7), and SMAD ubiquitination regulatory factor 2 (SMURF2) [37]. These results suggest that miR-145-5p might directly modulate KLF5 expression by post-transcriptional regulation via binding to its 3'-UTR or indirectly modulate KLF5 protein degradation by mediated E3 ubiquitin ligases. However, these speculations require further analysis.

There were several limitations in our study. First, it only explored the function of KLF5 in vitro. The application of the oncogenic function of the KLF5‐based strategy to develop antitumor therapeutics requires further validation by in vivo studies. Second, we have to collect more human NPC tissues to explore the correlation between miR-145-5p, KLF5 and FAK. It is challenging to target a transcription factor and therefore the targeting of newly identified downstream effectors, such as FAK in NPC cells, is preferred. FAK is a multi-functional regulator in several tumors, including colon, prostate, breast, thyroid, liver, gastric, and ovarian cancers [38]. The FAK signaling pathway has been studied in numerous tumors and has been found to regulate cellular proliferation, migration, invasion, and metastasis [39, 40]. However, the role of FAK in NPC is not clear. KLF5 contain three highly conserved and tandem zinc-finger motifs at their C-terminus, which bind to DNA GC or CACC boxes and regulate the transcription of downstream target genes [5, 41]. In Ingenuity Pathway Analysis, we found that KLF5 may directly or indirectly regulate several genes, which thereby modulate FAK expression. The regulation of FAK activity by miR-145-5p and KLF5 in NPC requires further exploration by molecular pathway prediction studies.

## Conclusions

Collectively, we report the novel role of miR-145-5p by regulating the KLF5/FAK pathway in NPC cells. Therefore, miR-145-5p-based therapeutics and FAK inhibitors could serve as potential therapeutic strategies for patients with NPC.

## Methods

### Reagents and antibodies

The compound ML264 was purchased from Sigma-Aldrich (SML1755), and its structure and synthesis pathway have been previously published. ML264 was dissolved in the Dimethyl sulfoxide (DMSO). DMSO was purchased from Sigma-Aldrich. Specific antibodies against E-cadherin (#3195), N-cadherin (#13116), phospho-FAK^Tyr397^ (#3283), FAK (#3285), PARP (#9532), Caspase 3 (#9662), α-tubulin (#3873) and β-actin (#3700) were purchased from Cell Signaling Technology, antibodies against KLF5 (TA324928) were purchased from OriGene, antibodies against Cyclin D1 (sc-753) and Cyclin B1 (sc-245) were purchased from Santa Cruz, and antibodies against P21 (#556430) were purchased from BD Pharmingen.

### Immunohistochemistry staining of KLF5

Nasopharyngeal carcinoma tissue array (NPC961) were purchased from Pantomics Inc. This tissue array had 50% missing rate, remaining 6 cases of primary tumors with paired normal tissues, 16 cases of primary tumors, 11 cases of lymph node metastatic tumors, 9 cases of normal/reactive nasopharyngeal mucosal tissues and one positive control. Precoated slides followed by deparaffinization, rehydration, and antigen retrieval as previously described [42]. Endogenous peroxidase was blocked per the manufacturer’s protocol (Dako, Carpinteria, CA). The slides were incubated with an anti-KLF5 polyclonal antibody (TA324928, Origene) at a 1: 800 dilution at room temperature for 1 h. Primary antibodies were detected using the Dako ChemMate EnVision Kit (K5001, Dako, Carpinteria, CA). Finally, the slides were counterstained with hematoxylin and investigated by light microscopy.

### Evaluation of immunohistochemical staining

Two qualified pathologists, who were blinded to the study, observed the immunohistochemical staining to classify the clinical status of the patients. The staining intensity (1, 2 or 3) was decided for each cell in a fixed field corresponding to the weak, intermediate and strong staining, respectively. The percentage of cells at each staining intensity expression was subsequently calculated, and H score (0–300) was assigned using the following formula: H score = 1 × (% of cells staining 1) + 2 × (% of cells staining 2) + 3 × (% of cells staining 3). H score for each case were calculated as the mean score of at least three individual section scores for each case, from which the mean score of all the individual field scores of each section was derived.

### Cell culture

Nasopharyngeal carcinoma cells line HONE-1 and NPC-TW01 were kindly provided by Dr. Chang-Shen Lin at Kaohsiung Medical University. NPC-TW03 and NPC-TW04 were gifts from Prof. Chi-Ying Huang (Institute of Biopharmaceutical Sciences, National Yang Ming Chiao Tung University, Taiwan). Four cell lines were cultured in Dulbecco's Modified Eagle Medium (DMEM, Gibco™, Thermo Fisher Scientific, MA, USA) supplemented with antibiotic–antimycotic (Gibco™, Thermo Fisher Scientific, MA, USA) and 10% fetal bovine serum (FBS, Gibco™, Thermo Fisher Scientific, MA, USA) at 37 °C in a 5% CO_2_ air atmosphere. NP69 human nasopharyngeal epithelial cell line was purchased from Sigma-Aldrich (SCC197). NP69 was cultured in keratinocyte serum-free medium (Gibco Cat. No. 10744019) supplemented with Keratinocyte-SFM Growth Supplement and 2% FBS.

### Cell transfection

NPC cells were transfected with synthetic hsa-miR-145-5p mimics (mirVana® miRNA mimics, Thermo Scientific™, MA, USA) to overexpress miR-145-5p. Scramble form of miRNA was used as a control (mirVana® miRNA mimics, Thermo Scientific™, MA, USA). KLF5 overexpression plasmids CMV-KLF5 were purchased from Sino Biological, Wayne, PA, USA. Cell transfections were performed using TurboFect (Thermo Scientific™, MA, USA) according to the manufacturer’s protocol.

### WST-1 cell proliferation assay

Cell proliferation was analyzed by Premix WST-1 Cell Proliferation Assay System (TaKaRa, Mountain View, CA, USA), at least, triplicate samples according to the manufacturer’s protocol. Briefly, HONE-1 (1 × 10^3^ cells/well), NPC-TW01 (1 × 10^3^ cells/well), NPC-TW03 (1.2 × 10^3^ cells/well) and NPC-TW04 (1.2 × 10^3^ cells/well) cells, after transfection, were seeded in 96-well plates in 100 μL medium and were incubated at 37 °C. After incubation for 24, 48, and 72 h, 10 µL of WST-1 solution was added to each well and incubated for 1 h. Finally, cell proliferation was analyzed by measuring absorbance at 450 nm in a spectrophotometer.

### BrdU ELISA cell proliferation assay

Cell proliferation was analyzed by Cell Proliferation ELISA, BrdU (colorimetric) (Roche, Basel, Switzerland), at least, triplicate samples according to the manufacturer’s protocol. Briefly, HONE-1 (1 × 10^3^ cells/well), NPC-TW01 (1 × 10^3^ cells/well), NPC-TW03 (1.2 × 10^3^ cells/well), NPC-TW04 (1.2 × 10^3^ cells/well) cells and NP69 (1.2 × 10^3^ cells/well) cells after transfection, were seeded in 96-well plates in 100 μL medium and were incubated at 37 °C. After incubation for 72 h, 10 µL of BrdU labeling solution was added to each well and incubated for 2 h at 37 °C. Removed labeling medium from adherent cells by tapping off and then added 200 μl/well FixDenat to each well and incubated for 30 min at room temperature. Next, removed FixDenat solution and added 100 μl/well Anti-BrdU-POD working solution and incubated for 90 min at room temperature. Removed antibody conjugate and rinsing wells three times with 200 to 300 μl/well Washing solution. Finally, added 100 μl Substrate Solution to each well, and cell proliferation was analyzed by measuring absorbance at 370 nm and 492 nm (reference wavelength) in a spectrophotometer.

### Wound-healing assay

Cell migration was estimated by wound-healing assay. Briefly, NPC and NP69 cells were counted and seeded in 2-well culture-inserts (ibidi, Gräfelfing, Germany) after transfection. After 24 h of incubation, the insert was removed to create the gap. The gap of NPC cells was maintained in DMEM medium with 1% FBS and then photographed at different time points using a phase contrast microscope. The wound healing area was analyzed by Image J software, and wound closure percentage was calculated as reported by Ayman Grada el. [43].

### Cell invasion assays

Cell invasion ability was measured by QCM ECMatrix cell invasion assay (Merck, Darmstadt, Germany) according to the manufacturer’s protocol. Briefly, NPC and NP69 cells were transfected and seeded (1.5 × 10^5^) into the upper chamber with serum-free medium and incubated onto the lower chamber having serum-containing medium for 24 h at 37 °C. The cells migrated through the ECM layer and clung to the bottom of the polycarbonate membrane. Invaded cells were incubated with cell detachment buffer, and then lysed and stained with CyQuant GR® dye (Merck, Darmstadt, Germany). Finally, the fluorescence was measured using a fluorescence plate reader through 480/520 nm filter set.

### Protein lysate preparation and Western blot analysis

The cell lines were plated in 6 cm dish using a density of 2 × 10^5^ cells and were allowed to grow to 60% confluence. Forty-eight hour after transfection of microRNA mimics, the cell lines were washed twice with cold PBS, lysed in cell lysis buffer (Cell signaling) for 10 min and scraped. The extracts were centrifuged at 13,500 g for 10 min at 4 °C. Protein concentrations were measured and equalized using Bio-Rad protein assay (Bio-Rad Laboratories) according to the manufacturer's instructions. Equivalent amounts of protein (30 μg) were then separated by SDS-PAGE and then transferred to polyvinylidene difluoride membranes (PVDF). In order to detect the protein expression of different molecules on the same membrane, the blots were cut prior to hybridization with antibodies. The PVDF membranes were blocked for 1 h in blocking buffer (1X Tris-buffered saline, 5% nonfat dry milk, and 0.1% Tween 20), which was subsequently replaced by the primary antibody in blocking buffer, overnight at 4 °C. After incubation, the membranes were washed three times in washing buffer (1X Tris-buffered saline and 0.1% Tween 20). Primary antibody was detected using AffiniPure Mouse or Rabbit Anti-Human IgG (Jackson ImmunoResearch, USA) and visualized with Immobilon™ Western Chemiluminescent HRP Substrate (Merck Millipore, USA). The images were acquired by ChemiDoc Touch Imager with Image Lab™ Software (BioRad). The Fig. 4a, 5b, c and 6d have been done in triplicate. The band quantification was performed with the ImageJ software and the data shown in the histograms represent the average of at least three independent experiments.

### RNA extraction and real-time PCR analyses

Total RNA was extracted from NPC cells using Trizol reagent (Invitrogen, Carlsbad, CA, USA) and then reverse transcribed to cDNA with reverse transcriptase and random primers. Real-time PCR was performed by StepOnePlus™ Real-Time PCR System (Applied Biosystems™, Thermo Fisher Scientific, MA, USA) using SYBR Green master mixture (Thermo Fisher Scientific, MA, USA). The following primers were used in the Real-Time-PCR experiment: KLF5 sense, 5'-CCCTTGCACATACACAATGC-3'; KLF5 antisense 5'- GGATGGAGGTGGGGTTAAAT-3'; GAPDH sense, 5'-GCACCACCAACTGCTTAGCA-3'; and GAPDH antisense, 5'-TCTTCTGGGTGGCAGTGATG-3'. The relative levels of KLF5 mRNA are expressed as the inverse log of the ΔΔCt value and normalized with the internal control gene, GAPDH [44].

### Quantitative real-time PCR (qRT-PCR)

Total RNA was extracted from NPC cells using Trizol reagent (Invitrogen, Carlsbad, CA, USA) and then reverse transcribed to microRNA with TaqMan™ MicroRNA Reverse Transcription Kit (Applied Biosystems™, Thermo Fisher Scientific, MA, USA) and random primers. Quantitative real-time PCR was performed by StepOnePlus™ Real-Time PCR System (Applied Biosystems™, Thermo Fisher Scientific, MA, USA) using TaqMan MicroRNA Assays (Catalog number: 4427975, Assay ID: 002,278, Applied Biosystems™, Thermo Fisher Scientific, MA, USA). The reactions were incubated at 95 °C for 20 s, followed by 45 cycles at 95 °C for 5 s and 60 °C for 35 s. An RT reaction containing a miRNA specific stem-loop reverse-transcription primer and a separate specific TaqMan miRNA Assay. The RT stem-loop primer provides the specificity for the mature miRNA target; it does not detect its precursor. The relative levels of hsa-miR-145-5p are expressed as the inverse log of the ΔΔC_t_ value and normalized with the internal control, U6.

### Dual-luciferase reporter assay

For dual-luciferase reporter assay, NPC cells were seeded in a 96-well plate and co-transfected with pmirGLO dual-luciferase miRNA target expression vector (catalog number: E1330, Promega, Madison, USA) using TurboFect reagent (Thermo Scientific™, MA, USA). The samples were wild-type and mutant KLF5, scramble control and miR-145-5p mimics. Renilla and Firefly luciferase activities were measured 48 h post-transfection using the dual-luciferase reporter assay kit (catalog number: E2920, Promega, Madison, USA) and Microplate Luminometer (BioTeck, Winooski, VT, USA).

### MiRNA target-predicting and bioinformatics

For predicting miRNA and mRNA direct interaction, we used miRNA target-predicting tools. The prediction tools contained Targetscan [45], miRDB [46], Miranda [47] and mirDIP [48]. To find potential transcription factor binding to FAK, we used PROMO tool to investigate putative transcription factor binding sites (TFBS) in FAK sequence [49]. To search potential molecular networks between KLF5 and FAK by Ingenuity Pathway Analysis software (QIAGEN Digital Insights).

### Statistical analysis

Experiments were performed independently at least three times. The results are shown as mean ± SD. All statistical analyses were performed using the SPSS 19.0 (IBM SPSS, Armonk, NY, USA) and Microsoft Office Excel for PC. Statistical significance of dissimilarity between groups was confirmed by two-tailed student's *t-test*. *P-*values less than 0.05 were regarded as statistically significant.

## Supplementary Information

**Additional file 1. **

## Data Availability

All data generated or analyzed during this study are included in this published article and its additional files.

## Abbreviations

KLF5: Krüppel-like factor 5
NPC: Nasopharyngeal carcinoma
FAK: Focal adhesion kinase
miRNAs: MicroRNAs
lncRNAs: Long non-coding RNAs
FBW7: F-box and WD repeat domain-containing 7
SMURF2: SMAD ubiquitination regulatory factor 2
IPA: Ingenuity Pathway Analysis
DMEM: Dulbecco’s Modified Eagle Medium
PVDF: Polyvinylidene difluoride membranes
