# Supplementary material for: MicroRNA-145-5p modulates Krüppel-like factor 5 and inhibits cell proliferation, migration, and invasion in nasopharyngeal carcinoma
Source: BMC Mol Cell Biol. 2022 Jul 14;23:28. doi: 10.1186/s12860-022-00430-9 (PMC9284881; doi:10.1186/s12860-022-00430-9)
Supplement: Supplementary file 1 — Additional file 1. [file 12860_2022_430_MOESM1_ESM.pdf]

## Full image of western blots combines with marker

**Fig. 2A**

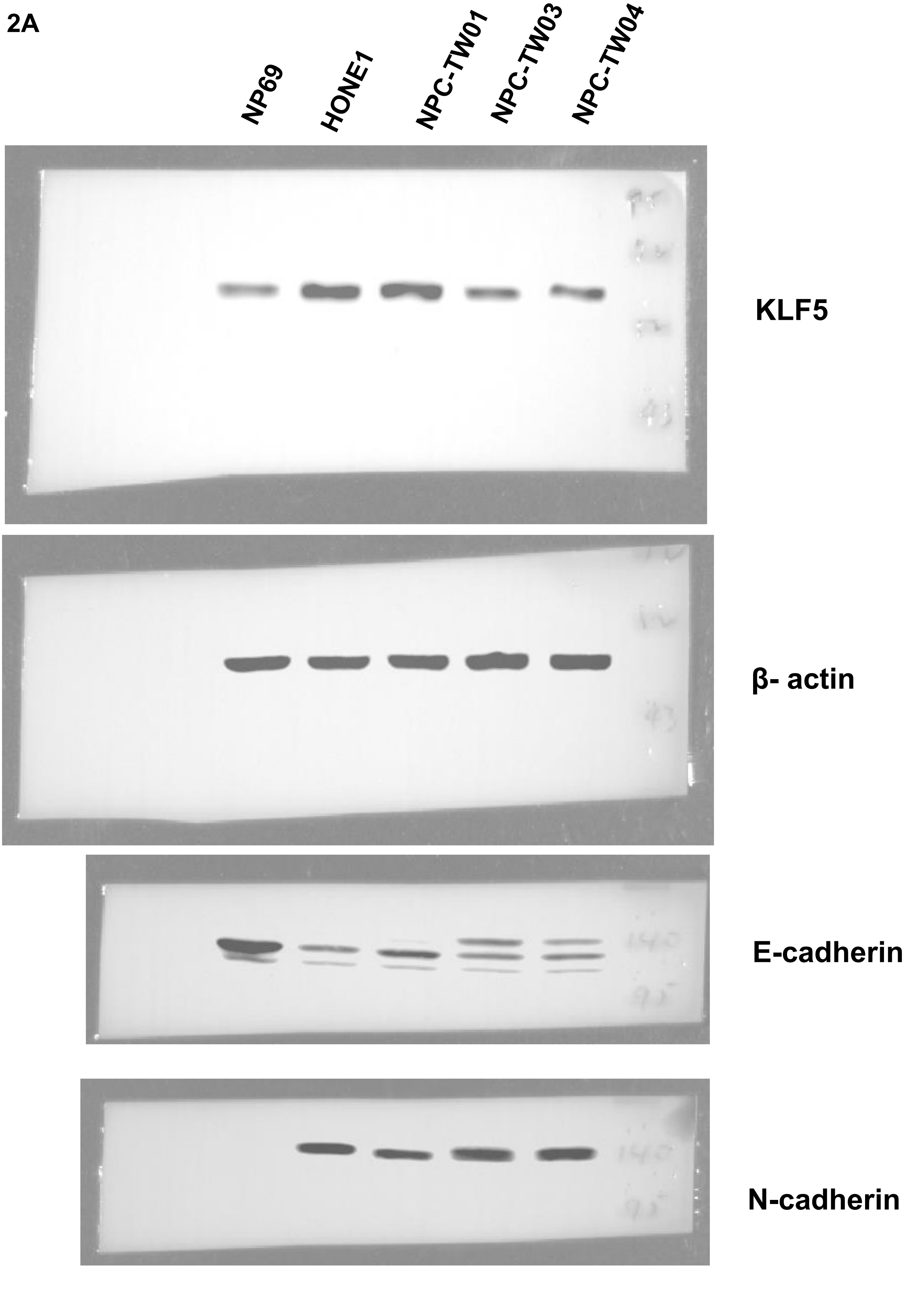

Fig. 2C

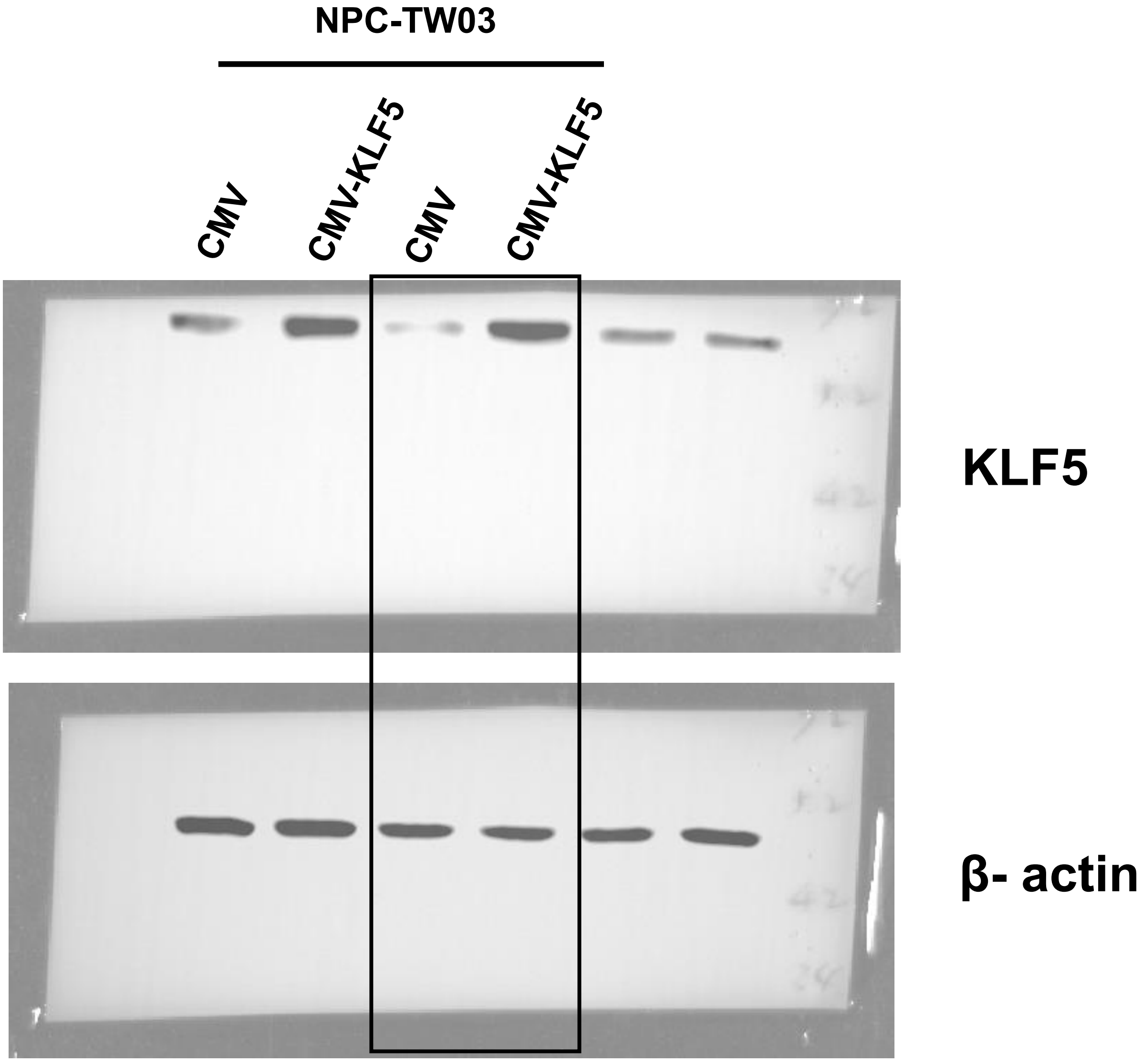

Fig. 2C

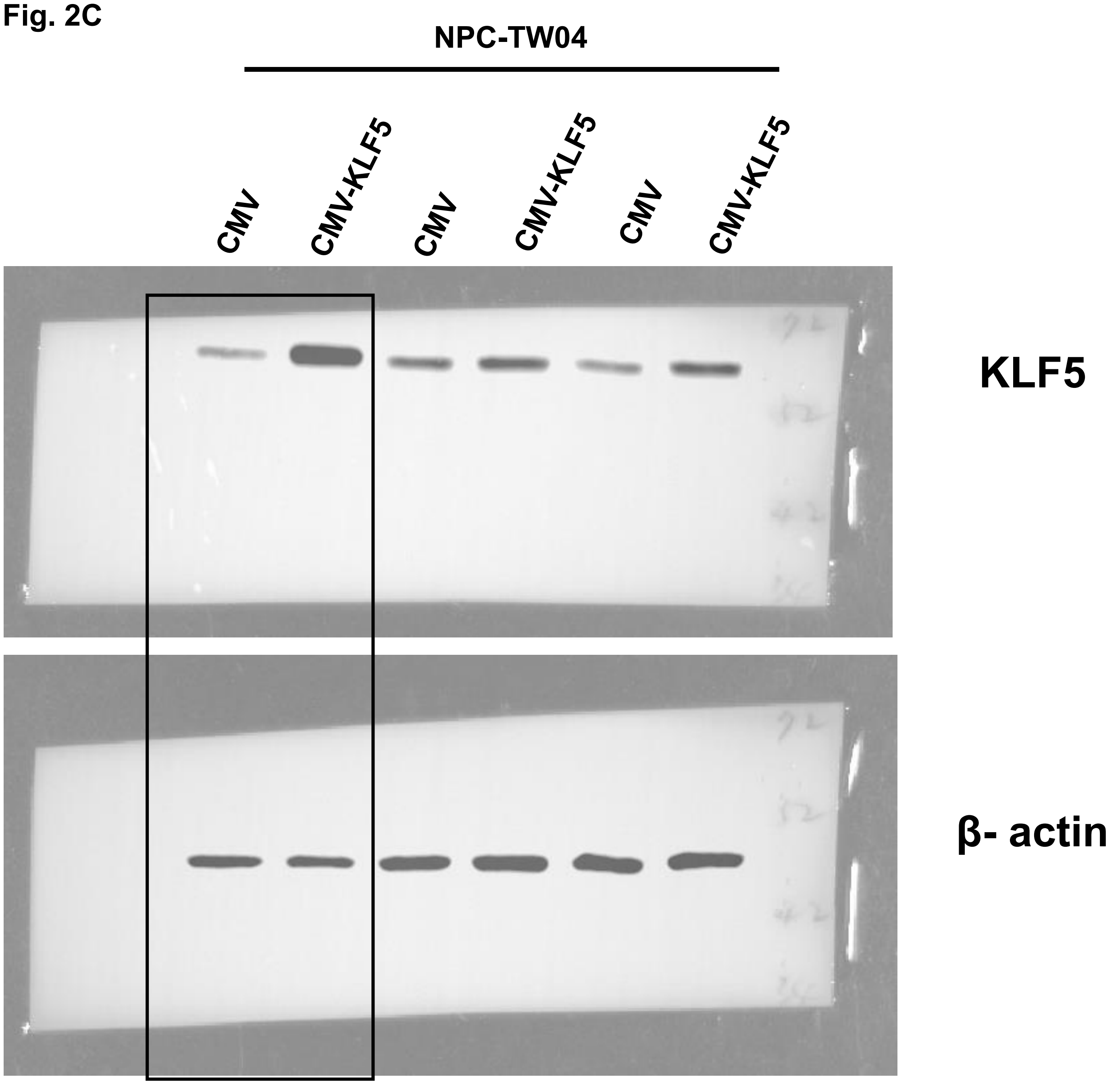

Fig. 4A

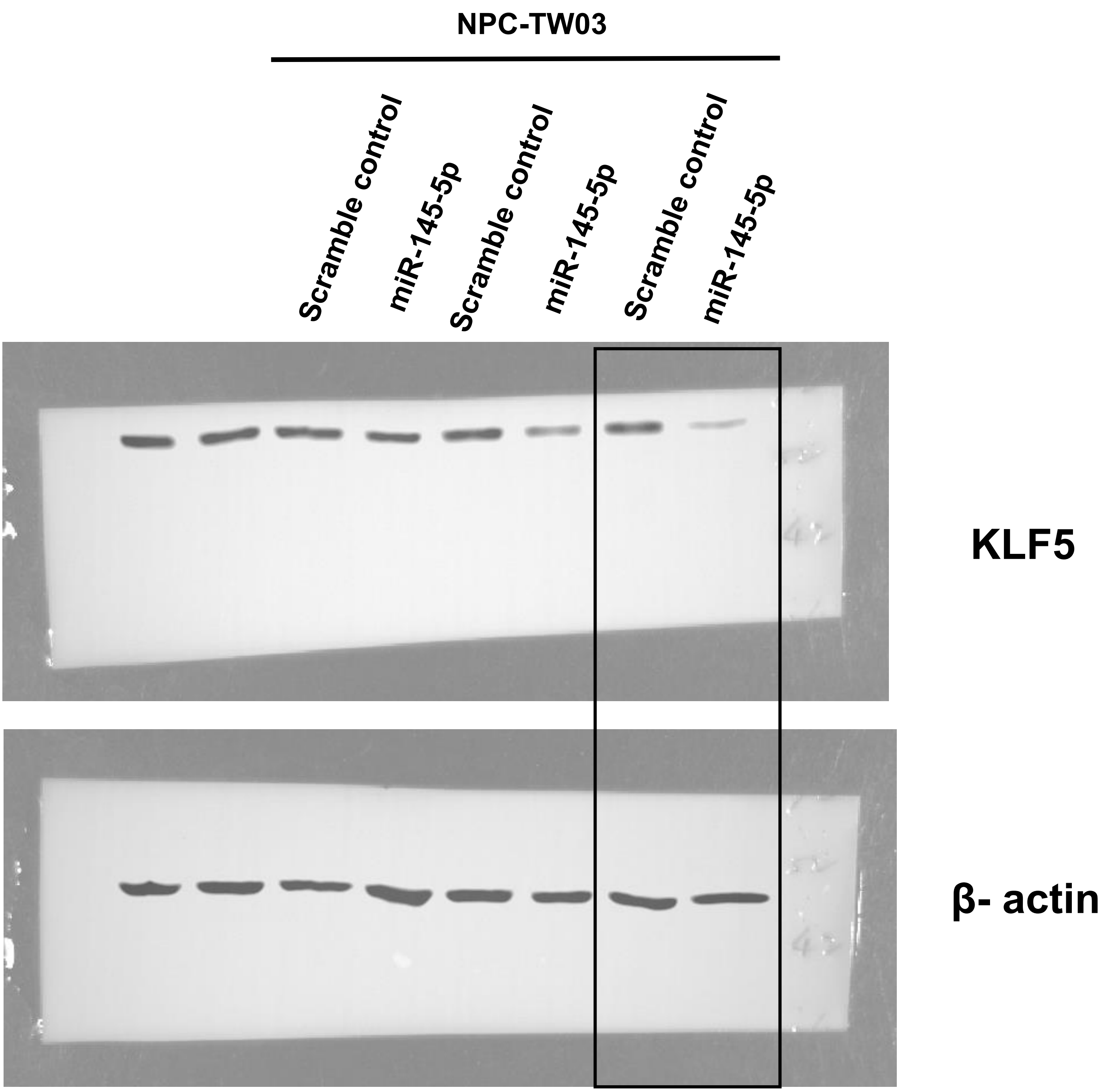
NPC-TW03
Scramble control
miR-145-5p
Scramble control
miR-145-5p
Scramble control
miR-145-5p
KLF5
β- actin

Fig. 4A

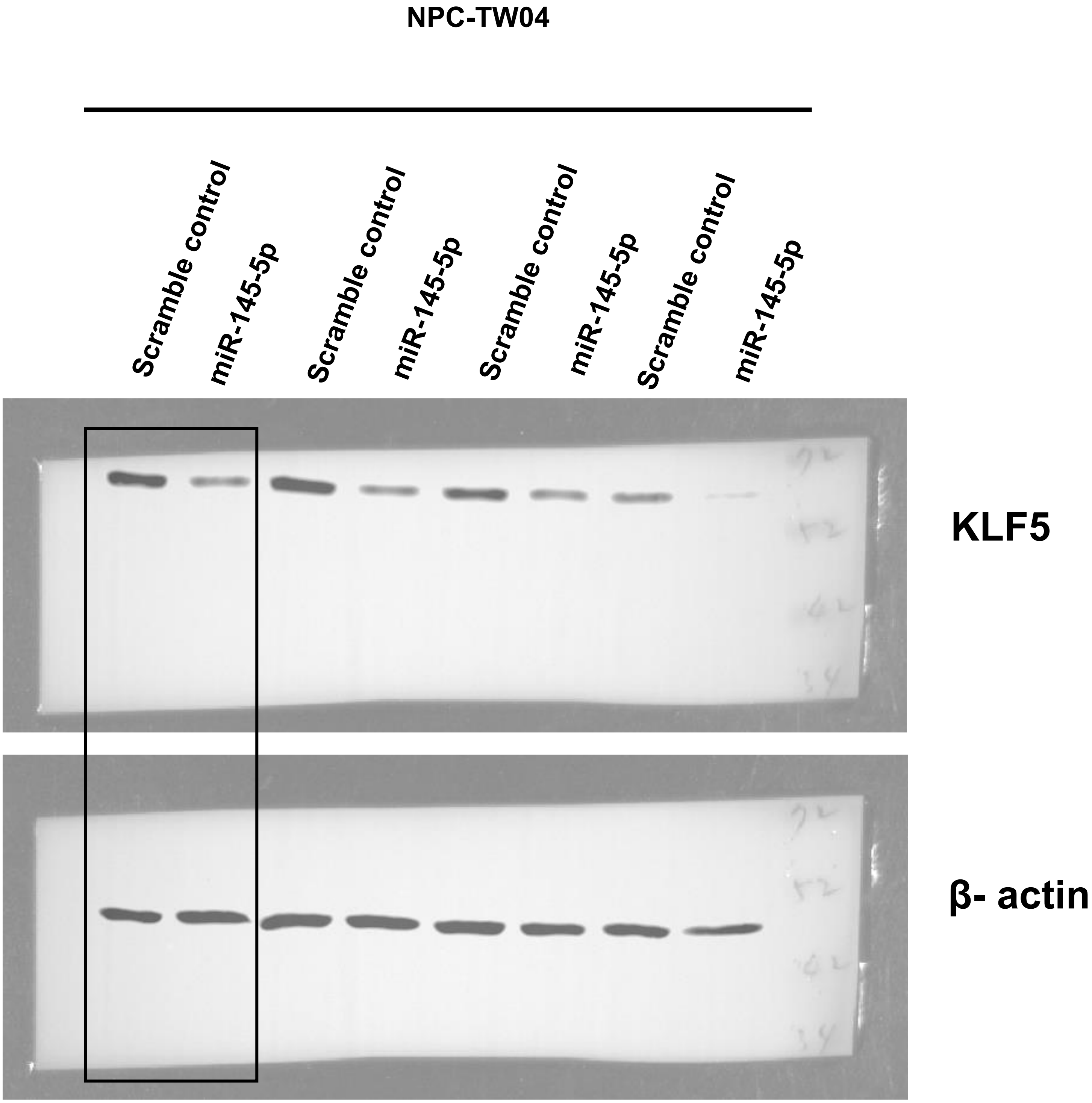
NPC-TW04
Scramble control
miR-145-5p
Scramble control
miR-145-5p
Scramble control
miR-145-5p
Scramble control
miR-145-5p
KLF5
β- actin

Fig. 5A

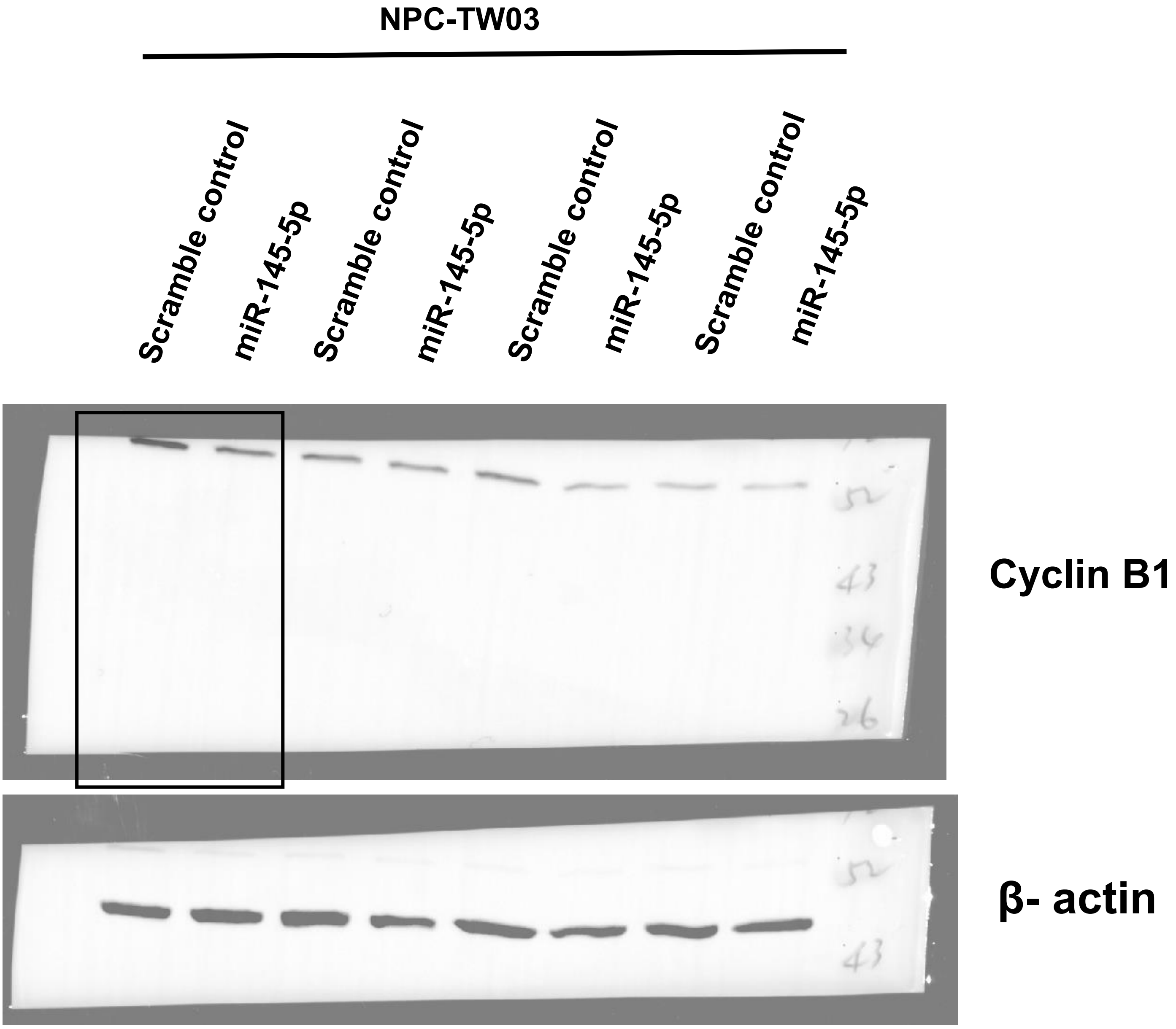
NPC-TW03
Scramble control
miR-145-5p
Scramble control
miR-145-5p
Scramble control
miR-145-5p
Scramble control
miR-145-5p
52
43
34
26
Cyclin B1
52
43
β- actin

Fig. 5A

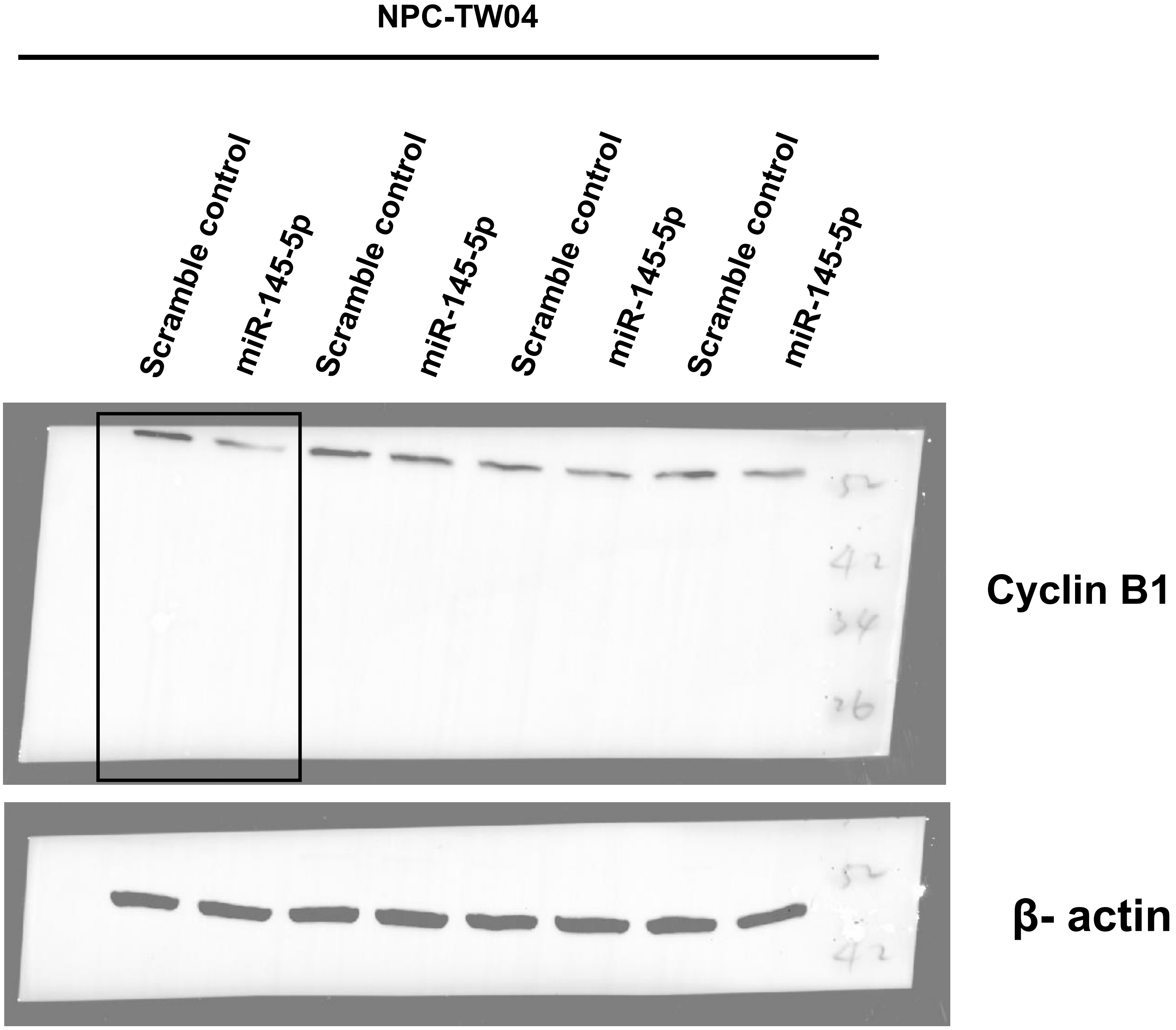
NPC-TW04
Scramble control
miR-145-5p
Scramble control
miR-145-5p
Scramble control
miR-145-5p
Scramble control
miR-145-5p
Cyclin B1
β- actin

Fig. 5A

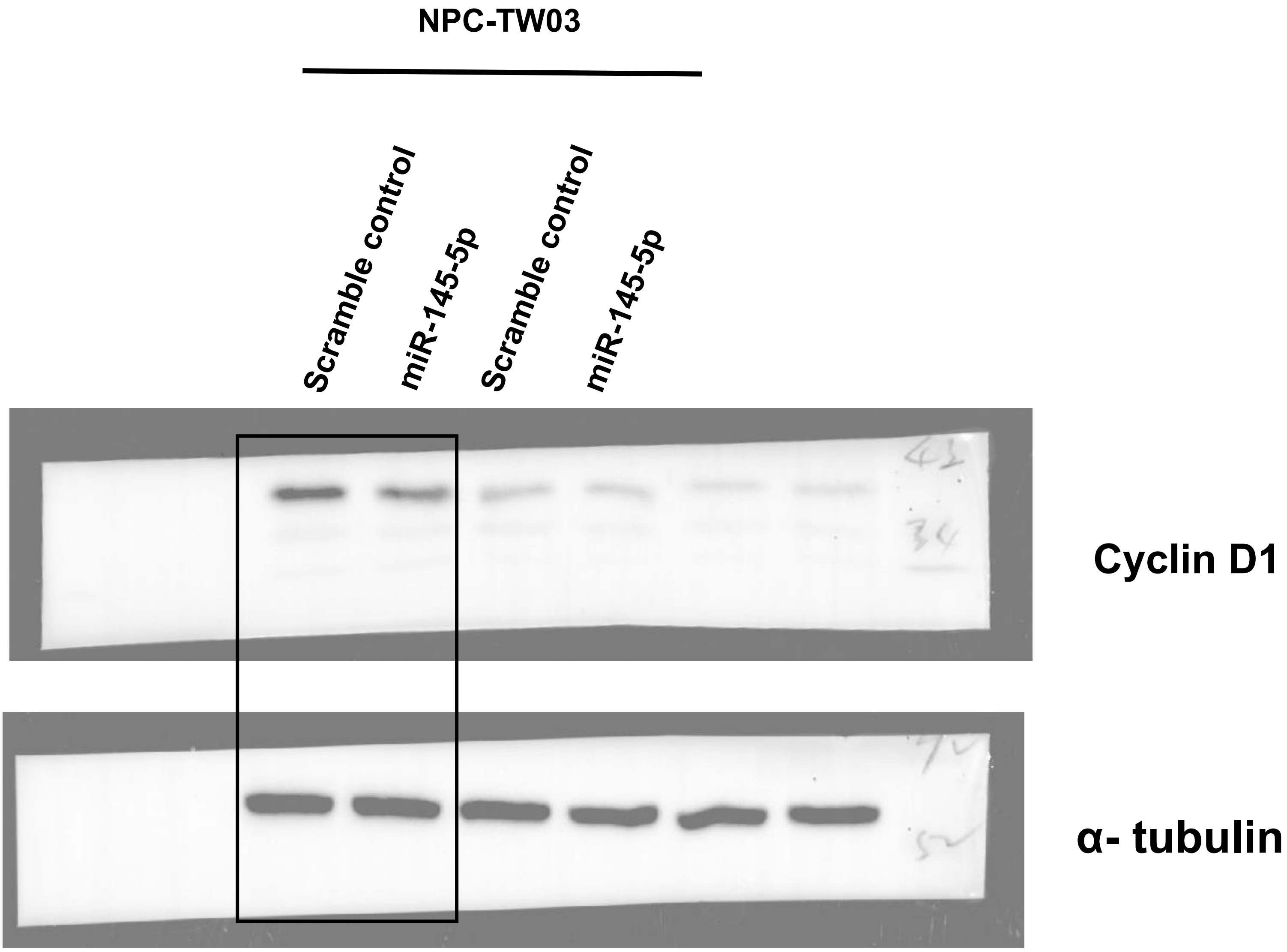
NPC-TW03
Scramble control
miR-145-5p
Scramble control
miR-145-5p
Cyclin D1
α- tubulin

Fig. 5A

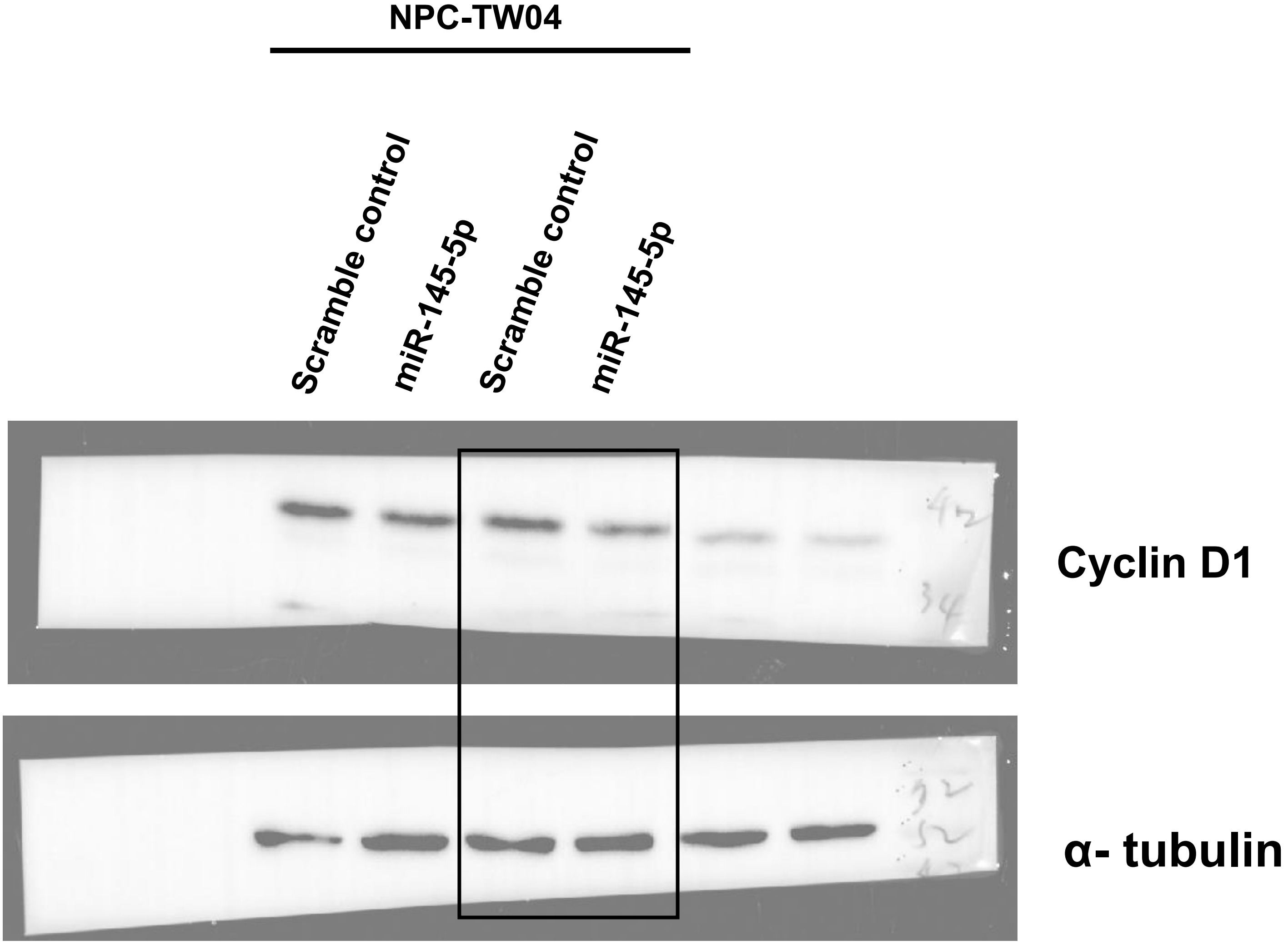
NPC-TW04
Scramble control
miR-145-5p
Scramble control
miR-145-5p
Cyclin D1
α- tubulin

Fig. 5A

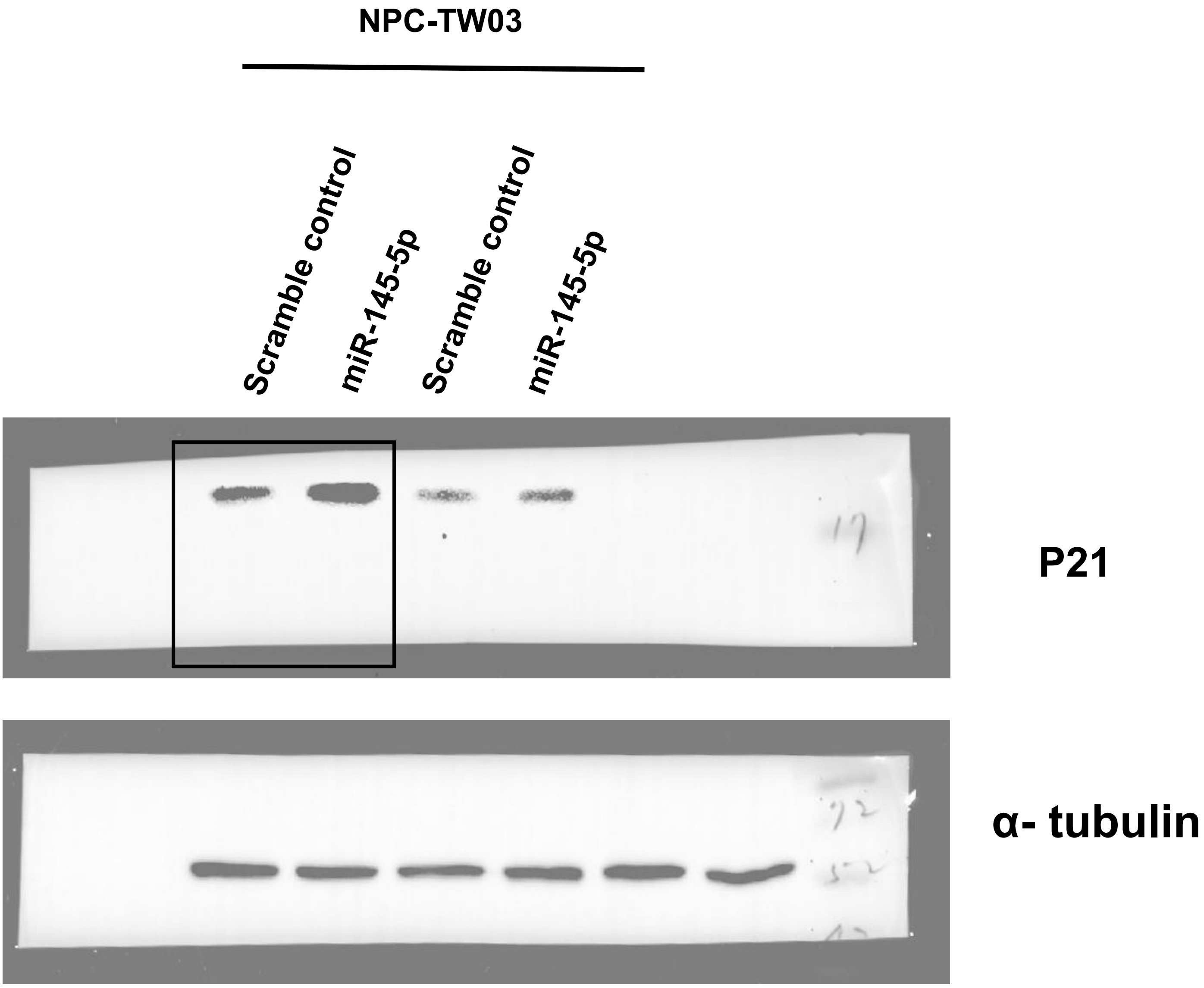
NPC-TW03
Scramble control
miR-145-5p
Scramble control
miR-145-5p
P21
α- tubulin

**Fig. 5A**

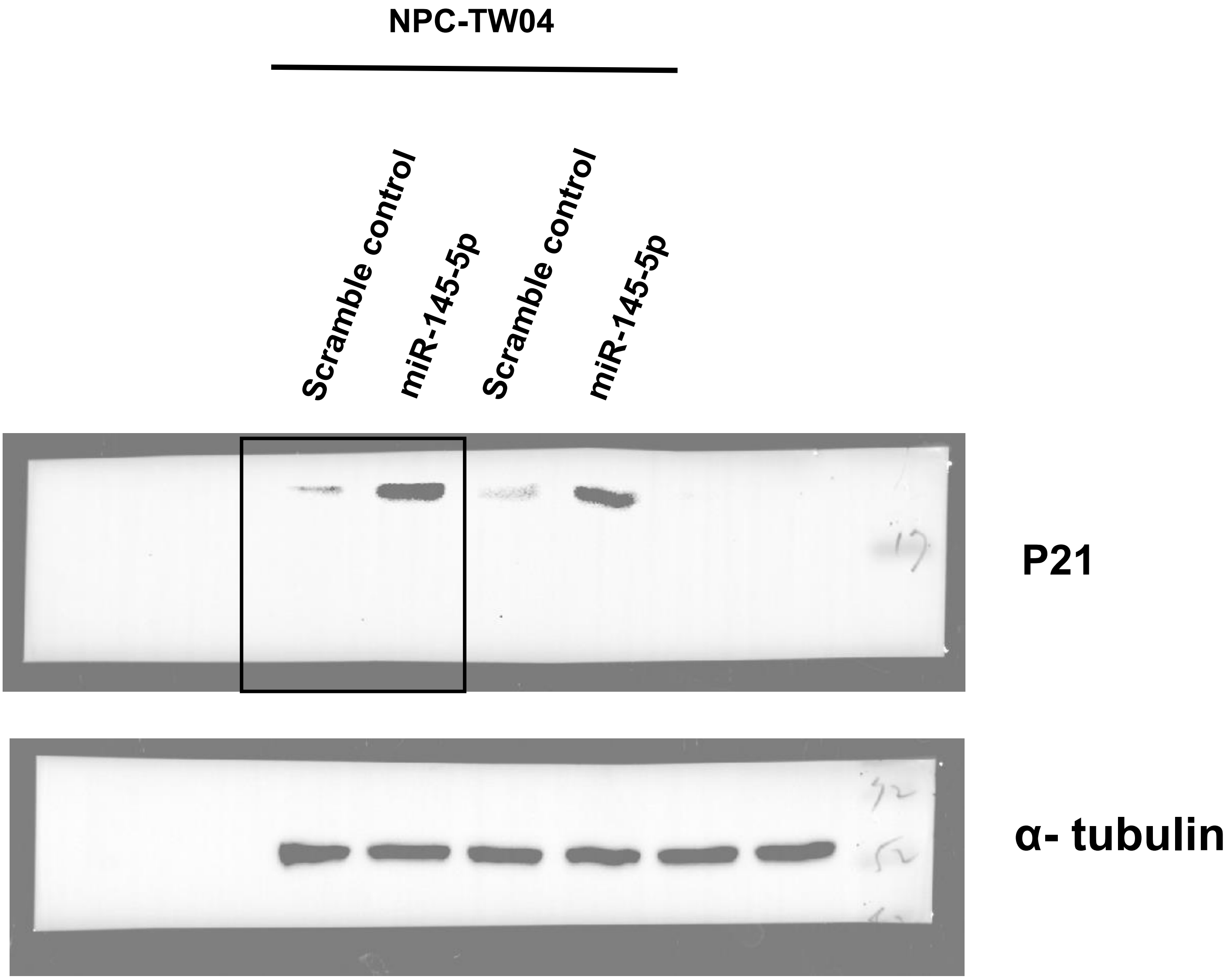
NPC-TW04
Scramble control
miR-145-5p
Scramble control
miR-145-5p
P21
α- tubulin

Fig. 5A

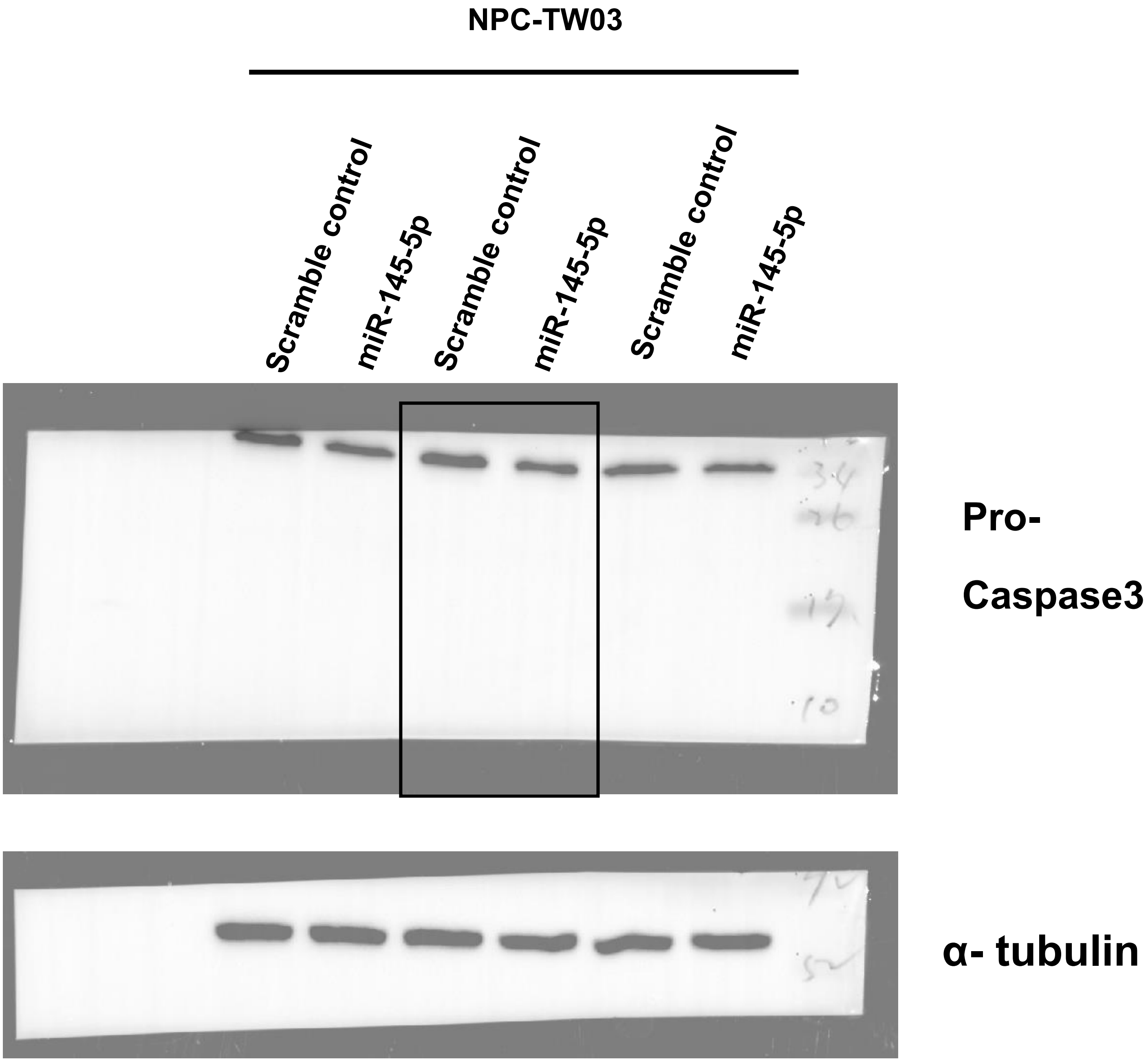
NPC-TW03
Scramble control
miR-145-5p
Scramble control
miR-145-5p
Scramble control
miR-145-5p
Pro-
Caspase3
α- tubulin

Fig. 5A

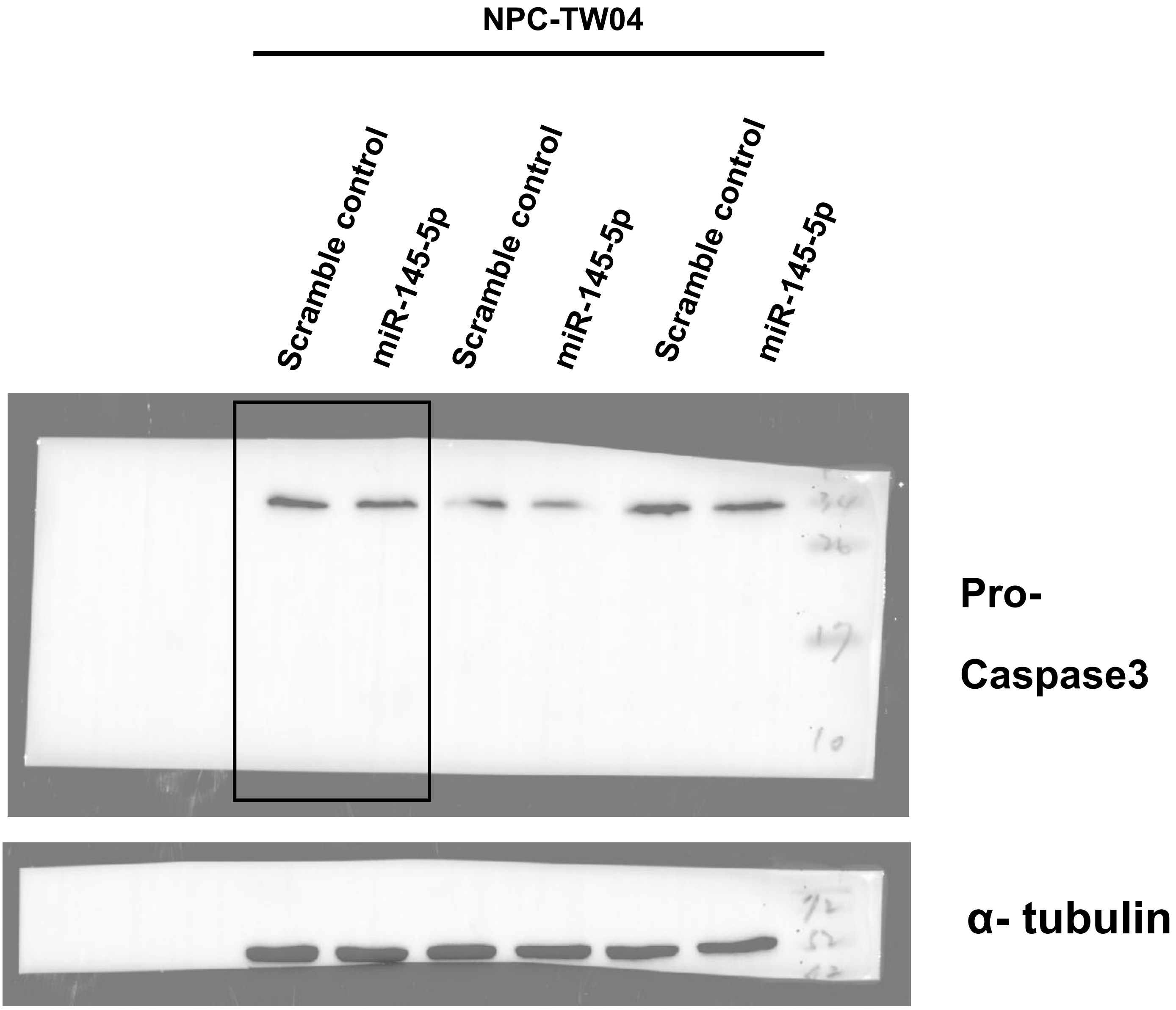
NPC-TW04
Scramble control
miR-145-5p
Scramble control
miR-145-5p
Scramble control
miR-145-5p
Pro-
Caspase3
α- tubulin

Fig. 5A

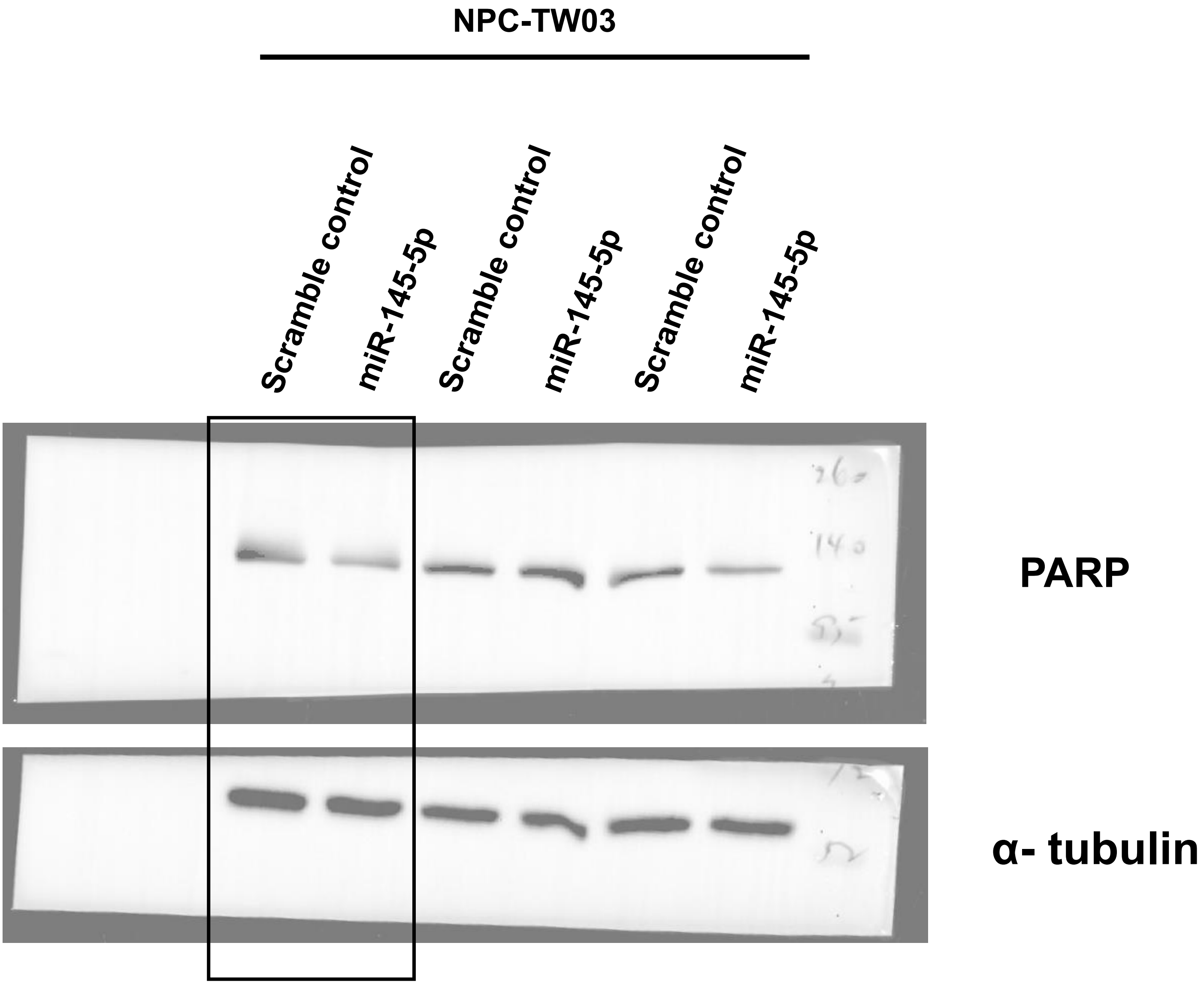
NPC-TW03
Scramble control
miR-145-5p
Scramble control
miR-145-5p
Scramble control
miR-145-5p
PARP
α- tubulin

**Fig. 5A**

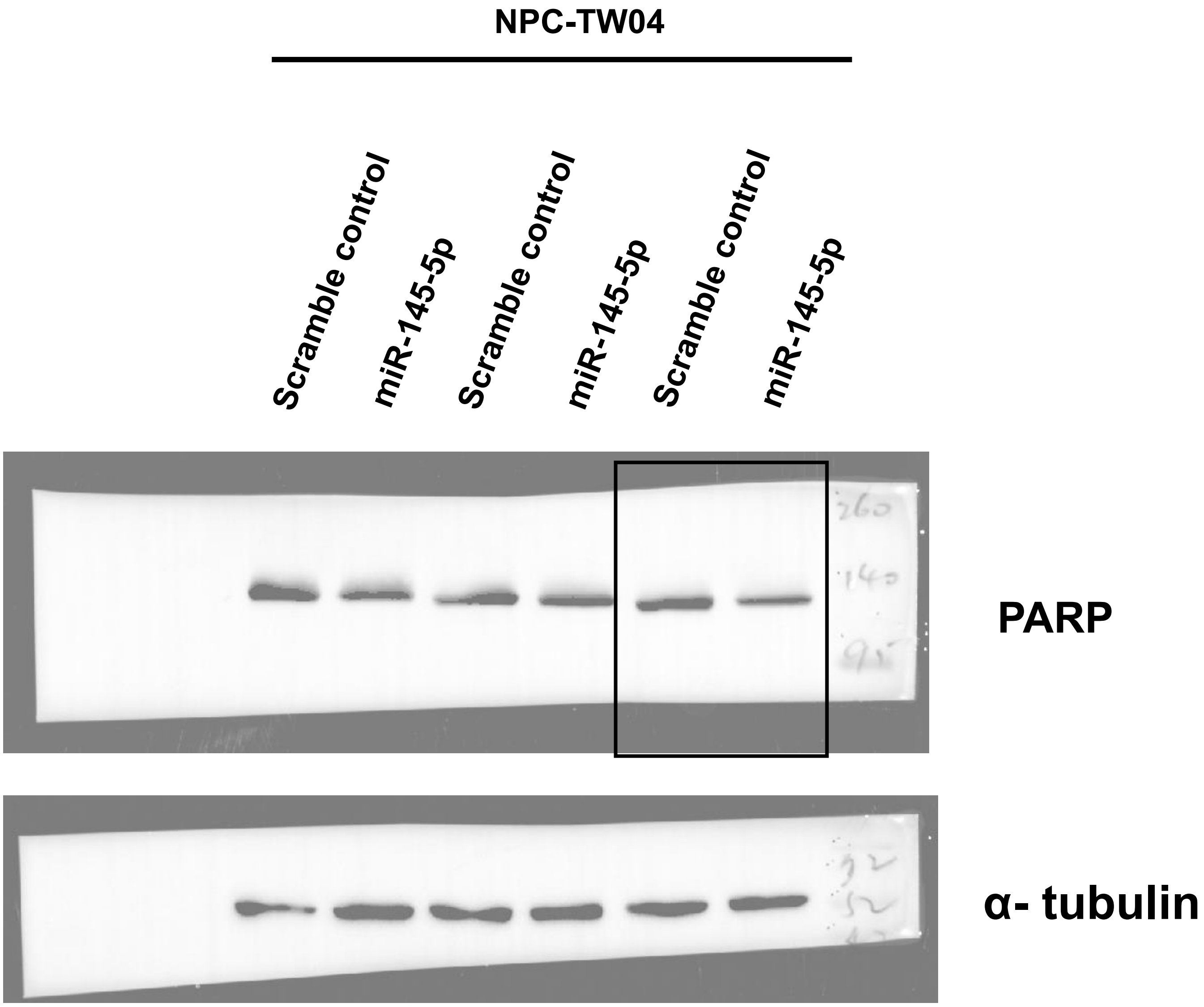
NPC-TW04
Scramble control
miR-145-5p
Scramble control
miR-145-5p
Scramble control
miR-145-5p
PARP
α- tubulin

Fig. 5B

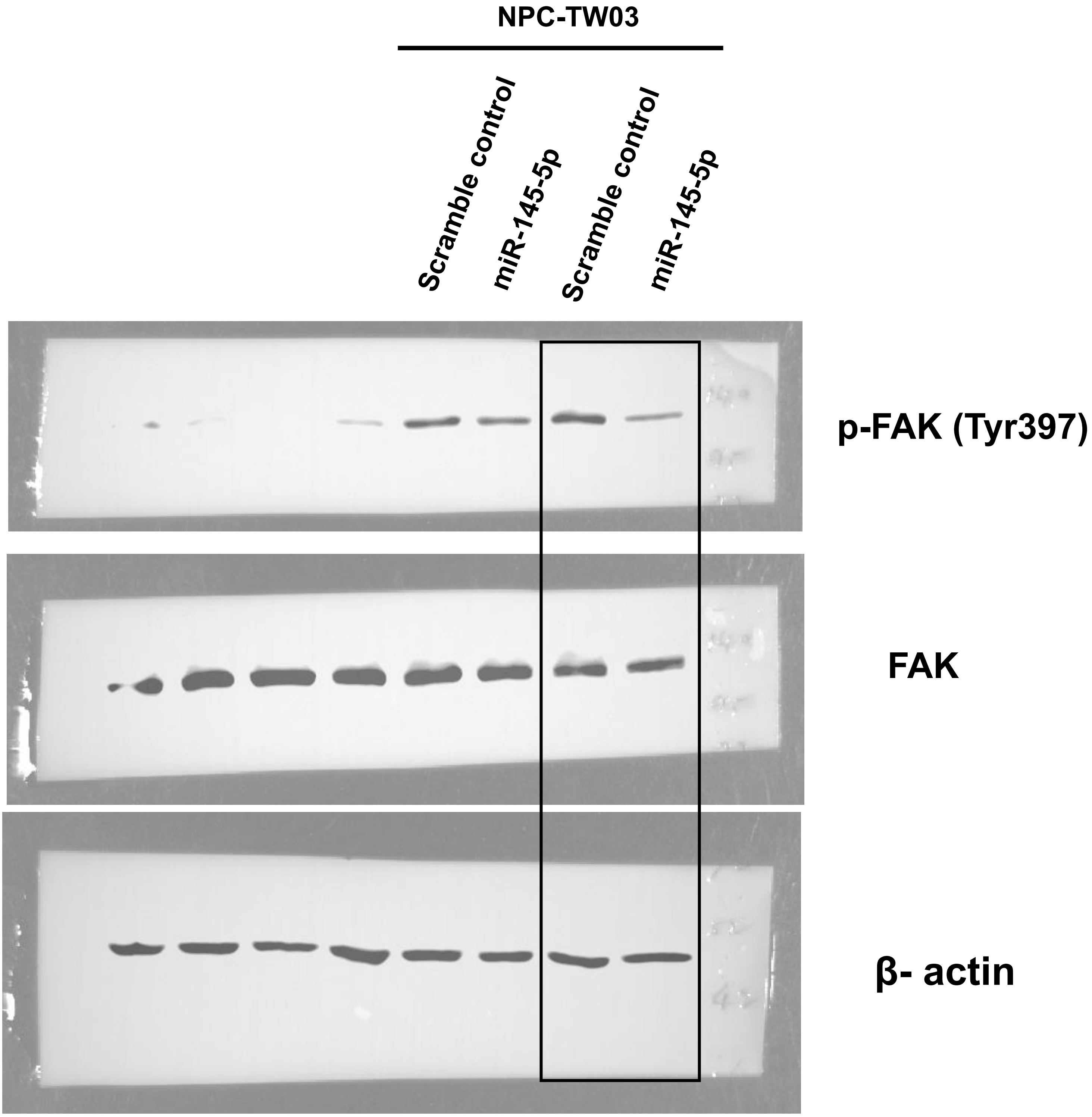
NPC-TW03
Scramble control
miR-145-5p
Scramble control
miR-145-5p
p-FAK (Tyr397)
FAK
β- actin

Fig. 5B

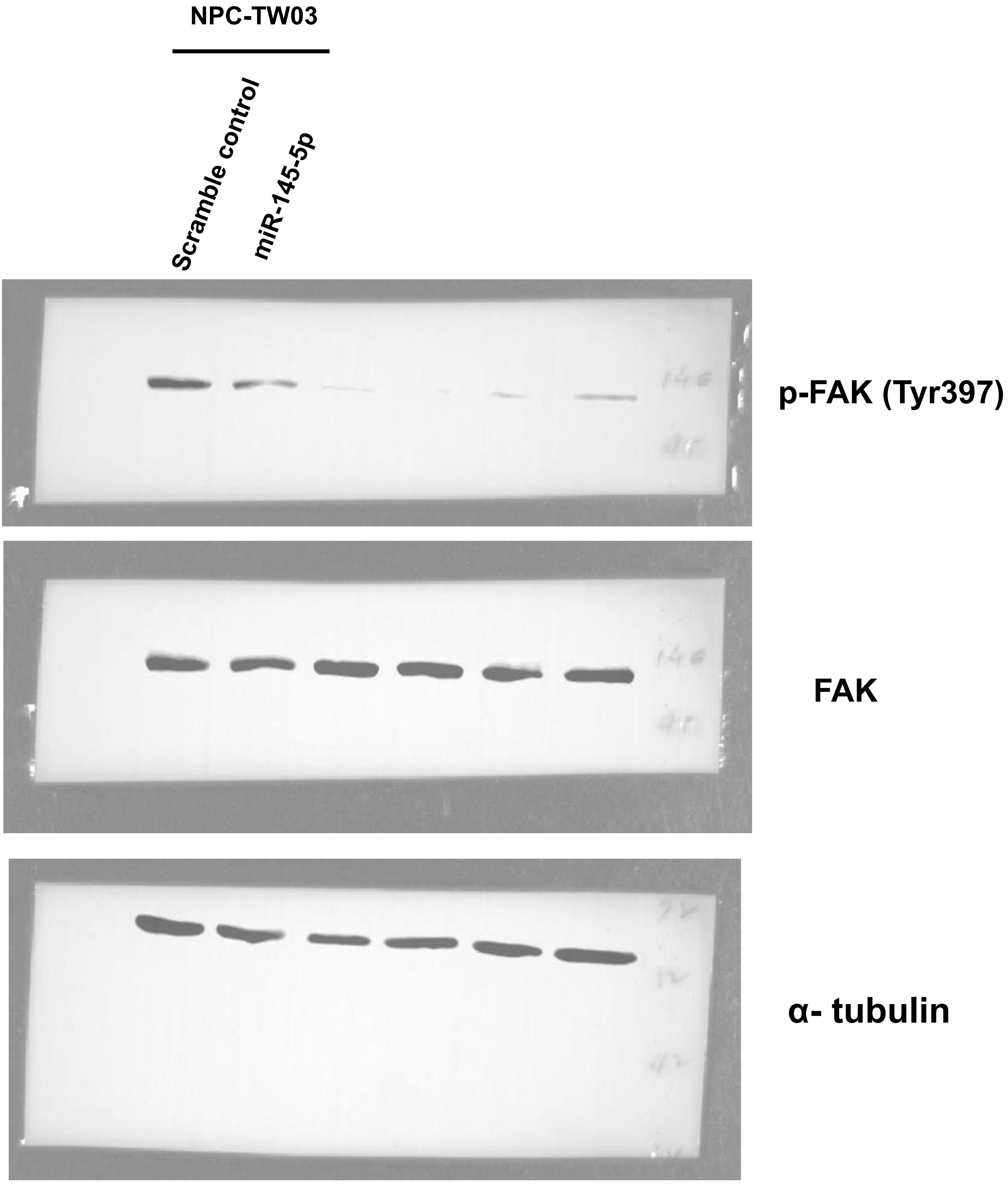
NPC-TW03
Scramble control
miR-145-5p
p-FAK (Tyr397)
FAK
α- tubulin

**Fig. 5B**

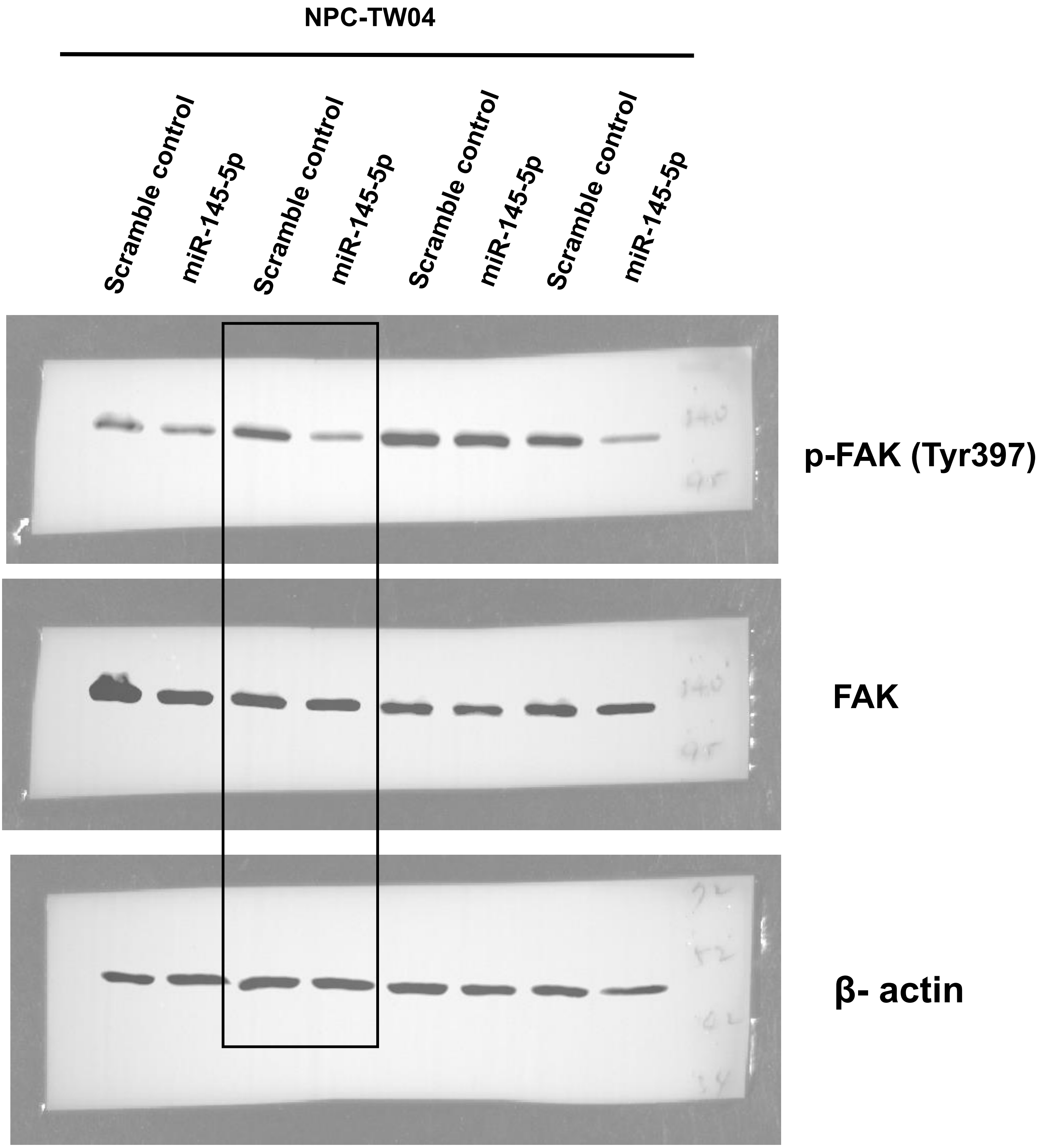
NPC-TW04
Scramble control
miR-145-5p
Scramble control
miR-145-5p
Scramble control
miR-145-5p
Scramble control
miR-145-5p
p-FAK (Tyr397)
FAK
β- actin

Fig. 5D

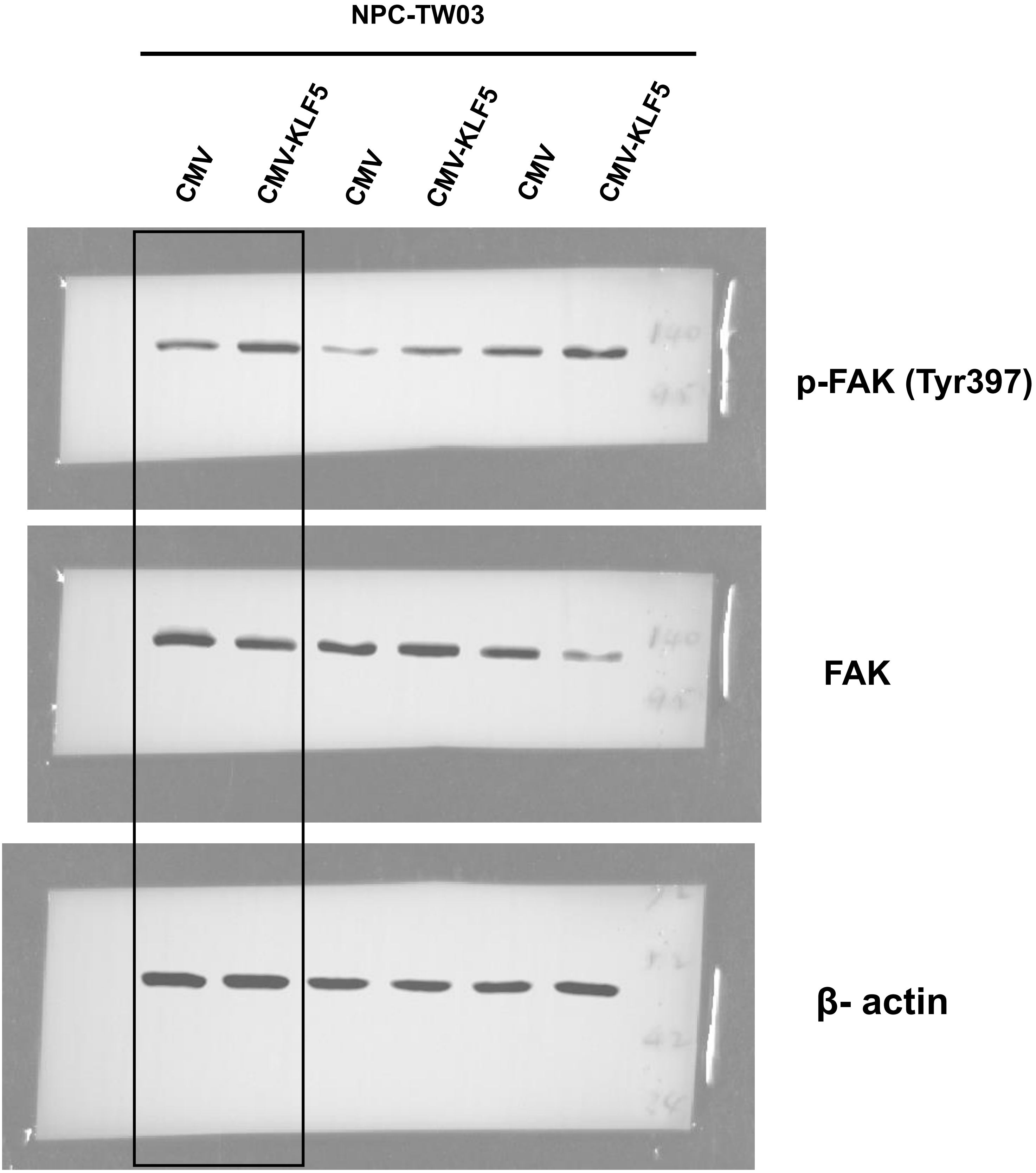
NPC-TW03
CMV
CMV-KLF5
CMV
CMV-KLF5
CMV
CMV-KLF5
p-FAK (Tyr397)
FAK
β- actin

**Fig. 5D**

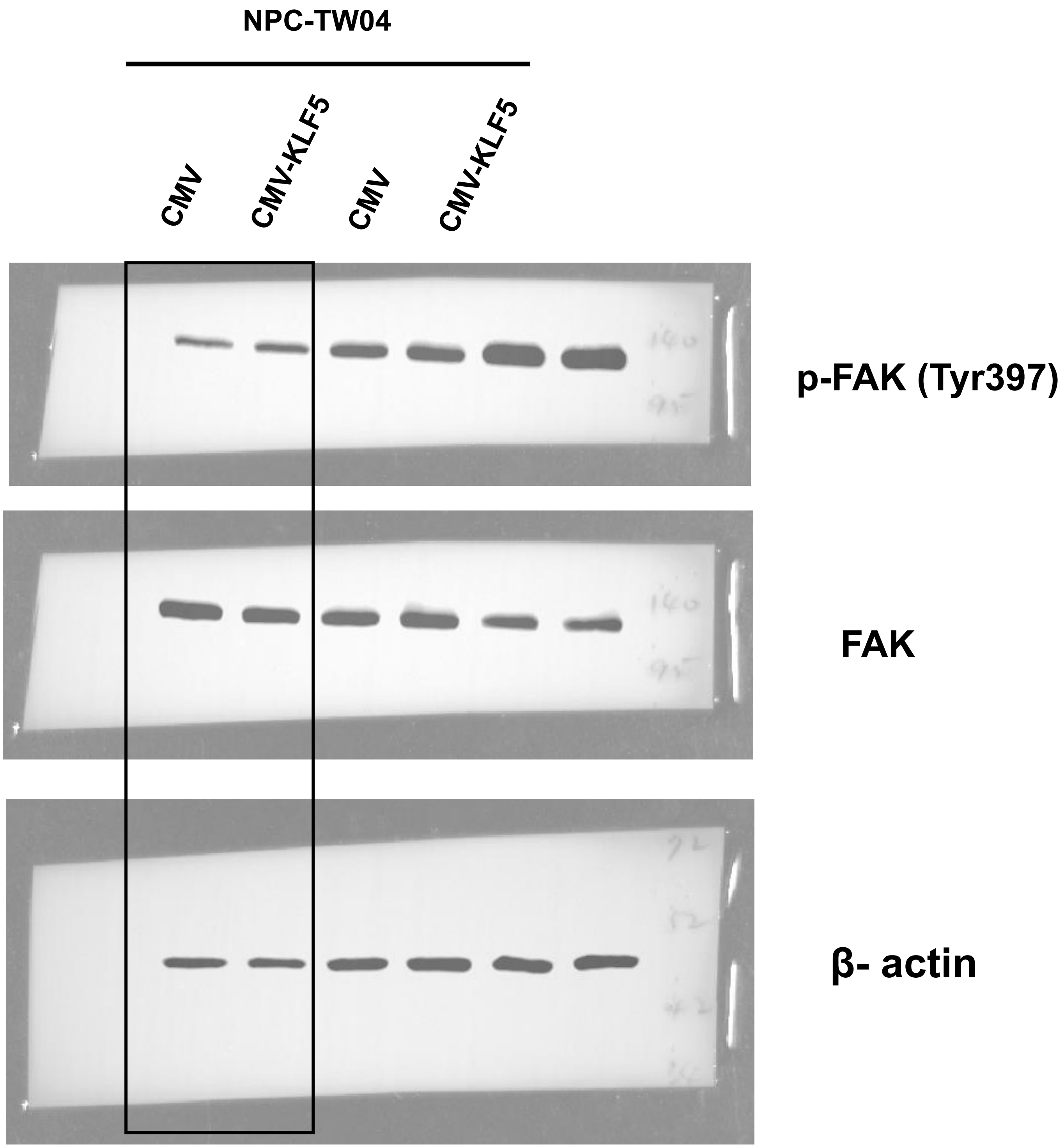

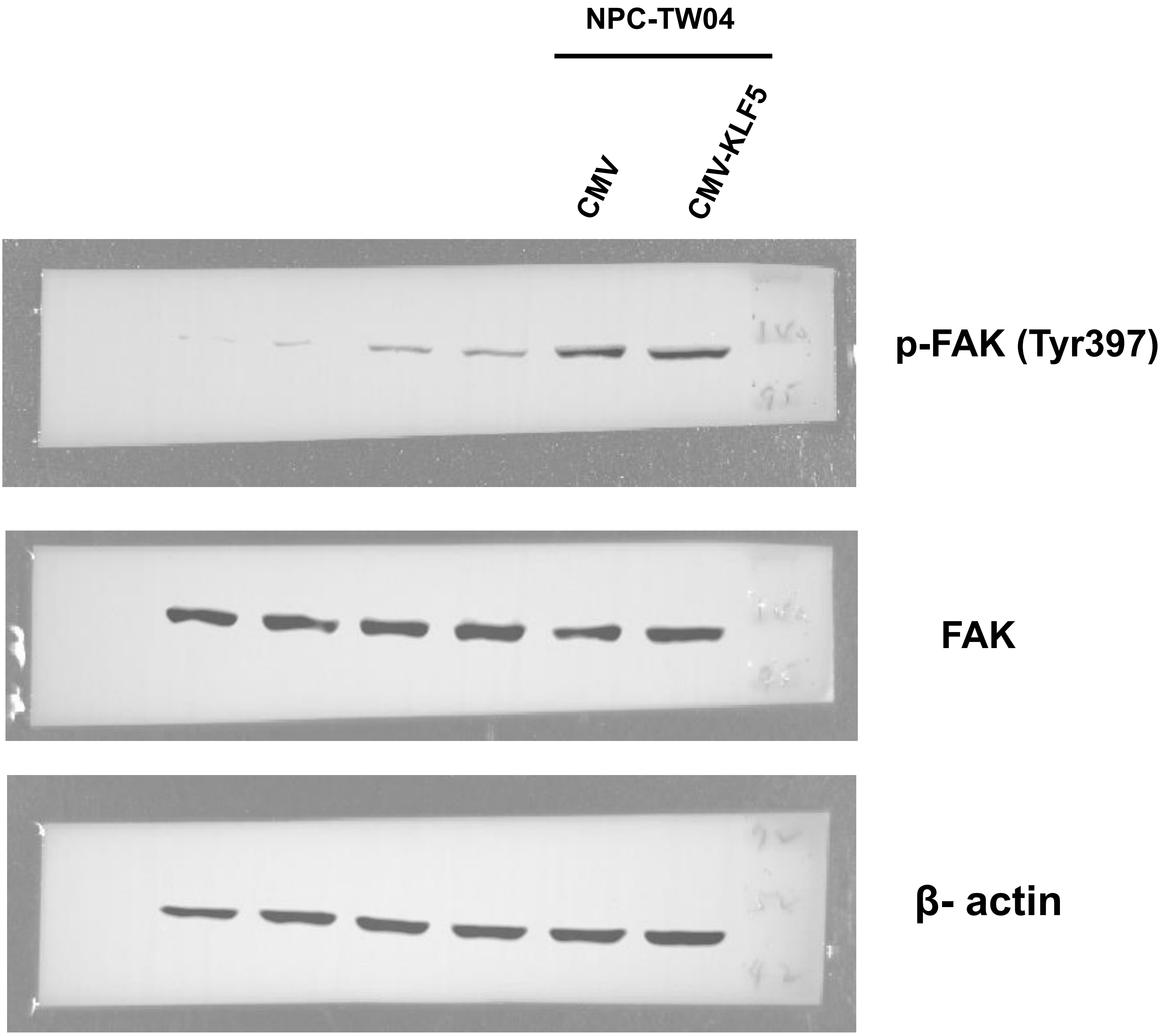

NPC-TW04
CMV
CMV-KLF5
p-FAK (Tyr397)
FAK
β- actin

**Fig. 6D**

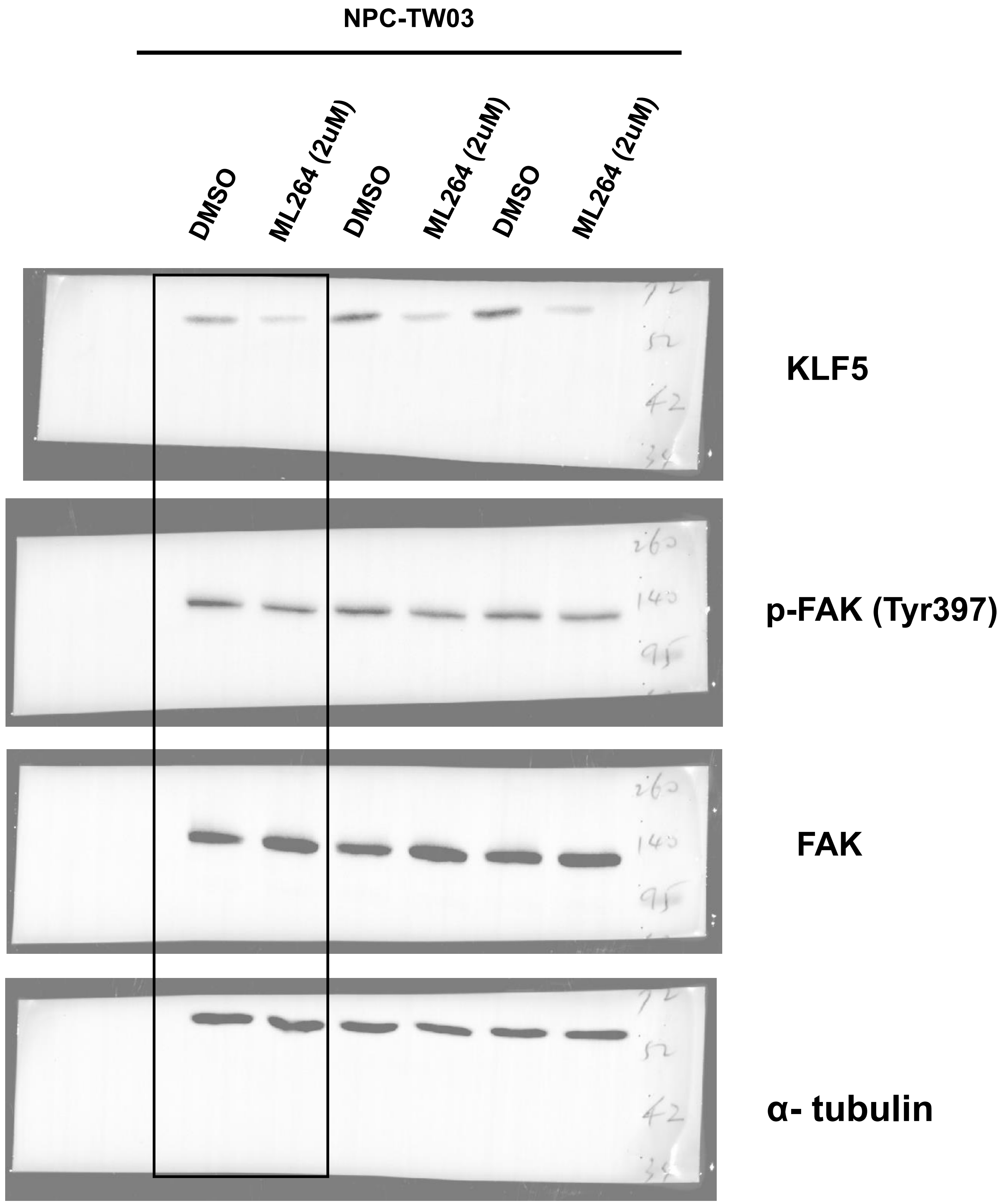
NPC-TW03
DMSO
ML264 (2uM)
DMSO
ML264 (2uM)
DMSO
ML264 (2uM)
KLF5
p-FAK (Tyr397)
FAK
α- tubulin

Fig. 6D

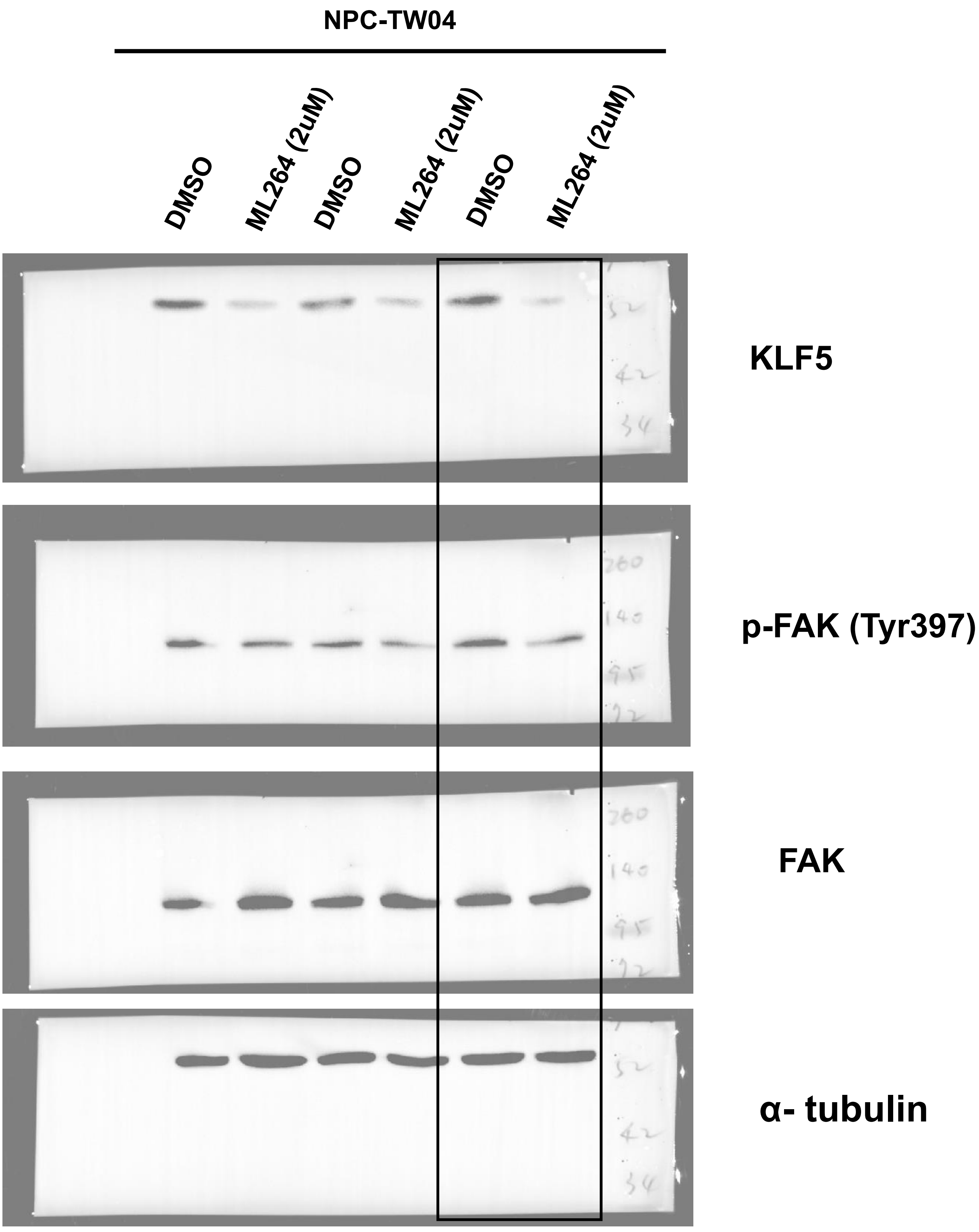
NPC-TW04
DMSO
ML264 (2uM)
DMSO
ML264 (2uM)
DMSO
ML264 (2uM)
KLF5
p-FAK (Tyr397)
FAK
α- tubulin
